# LncRNA MT1JP plays a protective role in intrahepatic cholangiocarcinoma by regulating miR-18a-5p/FBP1 axis

**DOI:** 10.1186/s12885-021-07838-0

**Published:** 2021-02-08

**Authors:** Wei Zhao, Jing Zhao, Xiao Guo, Yujie Feng, Bingyuan Zhang, Lantian Tian

**Affiliations:** 1 Department of Hepatopancreatobiliary Surgery, The Affiliated Hospital of Qingdao University, 1677 Wutaishan Road, Qingdao, 266000 People’s Republic of China; 2 Department of Pathology, The Affiliated Hospital of Qingdao University, Qingdao, 266000 People’s Republic of China; 3 Heart Center, Qingdao Fuwai Cardiovascular Hospital, Qingdao, 266034 People’s Republic of China

**Keywords:** lncRNA, MT1JP, Cholangiocarcinoma, miRNA, Tumor

## Abstract

**Background:**

Cholangiocarcinoma is a common malignant tumor of digestive system. LncRNA metallothionein 1 J, pseudogene (MT1JP) has been reported to play tumor-suppressing roles in multiple cancers. However, its effect on cholangiocarcinoma has not been evaluated.

**Methods:**

The expression of MT1JP in intrahepatic cholangiocarcinoma specimens and paired para-carcinoma tissues were detected by real-time PCR. The overexpression plasmid and siRNA of MT1JP were transfected into intrahepatic cholangiocarcinoma cells to change the expression levels of MT1JP. CCK-8, flow cytometry and transwell assays were performed to measure proliferation, cell cycle transition, apoptosis, migration and invasion. Dual-luciferase reporter assay, real-time PCR and western blot were carried out to screen the miRNA bound by MT1JP. In addition, xenograft experiment was used to determine the tumorigenesis of cholangiocarcinoma cells in nude mice.

**Results:**

MT1JP was downregulated in intrahepatic cholangiocarcinoma specimens, and its expression was related with TNM stage and lymph node metastasis. Overexpression of MT1JP inhibited proliferation, cell cycle transition, migration and invasion, and induced apoptosis in intrahepatic cholangiocarcinoma cells. The knockdown of MT1JP led to opposite results. MT1JP bound to miR-18a-5p to facilitate the expression of fructose-1,6-bisphosphatase 1 (FBP1). MiR-18a-5p was increased in intrahepatic cholangiocarcinoma samples, and its expression was negatively correlated with that of MT1JP. In addition, MT1JP also suppressed tumorigenesis in nude mice.

**Conclusions:**

MT1JP alleviated proliferation, migration and invasion, and induced apoptosis in cholangiocarcinoma cells by regulating miR-18a-5p/FBP1 axis. These findings may provide novel insights for clinical diagnosis and treatment of cholangiocarcinoma.

**Supplementary Information:**

The online version contains supplementary material available at 10.1186/s12885-021-07838-0.

## Background

Cholangiocarcinoma is a common malignant tumor of digestive system, and the overall incidence of cholangiocarcinoma has progressively increased worldwide over the past decades [1]. Based on anatomical location, cholangiocarcinoma is classified into intrahepatic, perihilar and distal cholangiocarcinoma [2]. The incidence of cholangiocarcinoma varies in different parts of the world. In Southeast Asia the morbidity of cholangiocarcinoma is as high as 0.113% in men and 0.05% in women, while in western countries the incidence is low at 0.0002% in men and 0.0001% in women [3, 4]. At present, surgery resection is still the only effective treatment for cholangiocarcinoma. As the anatomical concealment of bile duct, most patients with early stage are asymptomatic, and the early diagnose is difficult [5]. Most patients are diagnosed at an advanced stage with mestastasis [6]. For patients with advanced-stage or unresectable cholangiocarcinoma, the available systemic therapies are of limited effectiveness: the median overall survival with the current standard-of-care chemotherapy regimen is less than one year [7]. Therefore, the early diagnosis is vital for outcome of cholangiocarcinoma patients.

Long noncoding RNAs (lncRNAs) are a group of noncoding RNAs with length of more than 200 nucleotides. LncRNAs have been known abundant in lives, and to widely regulate gene expression through various mechanisms [8, 9]. LncRNA metallothionein 1 J pseudogene (MT1JP) was first reported as a tumor suppressor in liver cancer cells by regulating the translation of p53 [10]. Subsequently, MT1JP was found as a ceRNA to bind to miR-214-3p to facilitate expression of runt related transcription factor 3 (RUNX3), and suppressed cell proliferation, invasion and migration in gastric cancer cells [11]. The tumor-suppressing role of MT1JP has been verified in multiple cancer types, but its effect on cholangiocarcinoma has not been evaluated.

The TCGA database shows that MT1JP is downregulated in cholangiocarcinoma specimens, and its expression is positive correlated with that of fructose-1,6-bisphosphatase 1 (FBP1). FBP1 was found to restrain malignancies in cholangiocarcinoma cell in our previous study [12]. Through bioinformatics website analysis, MT1JP was predicted to be bound by miR-18a-5p seed sequence, which also bound to FBP1 3′ untranslation region (UTR). Because MT1JP has previously been reported to act as a miRNA sponge, we hypothesized that MT1JP functioned by sponging miR-18a-5p and regulating FBP1 expression in cholangiocarcinoma. The present study aimed to verify the hypothesis and investigated the effect of MT1JP in cholangiocarcinoma cells via gain- and loss-of-function experiments.

## Methods

### Clinical specimens

Twelve paired of intrahepatic cholangiocarcinoma and para-carcinoma tissues were collected from January 2016 to December 2019 in hepatological surgery department, affiliated hospital of Qiangdao Unversity via surgery resection. The patients had not received chemotherapy or radiotherapy treatment. The specimens were identified as cholangiocarcionma by pathological examination. Informed consent was obtained from each patient. The specimen collection and experimental procedure were line with Declaration of Helsinki, and approved by Ethics Committee of Affiliated Hospital of Qingdao University.

The expression levels of MT1JP and miR-18a-5p in the cholangiocarcinoma specimens were detected with real-time PCR.

The expression level of MT1JP in another thirty intrahepatic cholangiocarcinoma samples was analyzed with the patients’ clinicalpathological characteristics by Chi-square test.

### Real-time PCR

Total RNA was extracted with TRIpure reagent (BioTeke, Beijing, China), and reversely transcribed into cDNA with M-MLV reverse transcriptase (BioTeke), in presence of Olig (dT) and random, or specific miRNA primers (GenScript, Nanjing, China). The cDNA was used for real-time PCR to detect the expression levels of MT1JP and miR-18a-5p, and mRNA level of FBP1 with 2 × Power Taq PCR MasterMix (BioTeke) and SYBR Green (Sigma, St. Louis, USA). The PCR was performed with an Exicycler™ 96 V4 Real-Time Quantitative Thermal Block (Bioneer, Daejeon, Korea). The data were analyzed using 2^-ΔΔCt^ method. β-actin served as the internal control of MT1JP, and 5S served as the internal control of miR-18a-5p. The sequence information of primers was shown in Table 1.

**Table 1:** The sequence information of primers used in this study

Name	Sequence (5′-3′)
MT1JP Forward	GAAATGGACCCCAACTACTC
MT1JP Reverse	GTTCCCACATCAGGCACAGC
β-actin Forward	CACTGTGCCCATCTACGAGG
β-actin Reverse	TAATGTCACGCACGATTTCC
hsa-miR-18a-5p RT	GTTGGCTCTGGTGCAGGGTCCGAGGTATTCGCACCAGAGCCAACCTATCT
5S RT	GTTGGCTCTGGTGCAGGGTCCGAGGTATTCGCACCAGAGCCAACAAAGCCTAC
hsa-miR-18a-5p Forward	TAAGGTGCATCTAGTGCAGATAG
hsa-miR-18a-5p Reverse	GCAGGGTCCGAGGTATTC
5S Forward	GATCTCGGAAGCTAAGCAGG
5S Reverse	TGGTGCAGGGTCCGAGGTAT

### Western blot

The protein was extracted using lysis buffer supplemented with 1 mM PMSF (Beyotime, Haimen, China), and the concentration was determined with BCA protein assay kit (Beyotime). Then the protein was separated with SDS-PAGE, and transferred onto polyvinylidene difluoride membrane (Millipore, Boston, USA). The membrane was blocked with skim milk (YILI, Hohhot, Inner Mongolia, China) at room temperature for 1 h, and incubated with following antibodies at 4 °C overnight: rabbit anti-proliferating cell nuclear antigen (PCNA) (1:1000; cat. no. A0264, Abclonal, Wuhan, Hubei, China), rabbit anti-caspase-3 (1:1000; cat. no. A19654, Abclonal), rabbit anti-poly ADP-ribose polymerase (PARP) (1:1000; cat. no. A0942, Abclonal), rabbit anti-cyclin E (1:1000; cat. no. AF0144, Affinity, Changzhou, Jiangsu, China), rabbit anti-cyclin B1 (1:1000; cat. no. A2056, Abclonal), rabbit anti-fructose-1,6-bisphosphatase 1 (FBP1) (1:5000; cat. no. 12842–1-AP, Proteintech, Wuhan, Hubei, China) or mouse anti-β-actin (1:1000; cat. no. sc-47,778, Santa Cruz, USA). After rinsing with TBST, the membrane was incubated with goat anti-rabbit or mouse secondary antibody (1:5000; cat. no. A0208, A0216, Beyotime) at 37 °C for 45 min. The protein in the membrane was reacted with ECL reagent (Beyotime), followed with a signal exposure in the dark. The optical density of the blotting bands was analyzed with Gel-Pro-Analyzer software.

### Cell culture

Primary intrahepatic cholangetic epithelial cells were purchased from iCell (Shanghai, China), and cultured with primary epithelial cell medium (iCell) at 37 °C with 5% CO_2_. The primary intrahepatic cholangeitc epithelial cells were identified by immunofluorescent staining of cytokeratin 19 (CK19), and the results were shown in Fig. S12. The cell culture and identification was consistent with our previous reports [12].

Cholangiocarcinoma cell lines HCCC-9810 and RBE were purchased from Procell (Wuhan, China), and HUCCT1 from Zhongqiaoxinzhou (Shanghai, China). The cells were cultured in RPMI-1640 medium (Gibco BRL, Gaithersburg, USA) supplemented with 10% fetal bovine serum (FBS) (Hyclone, Logan, USA) at 37 °C with 5% CO_2_. The cell lines have been authenticated by STR, and test as mycoplasma negative.

Cell transfection was performed with Lipofectamine 2000 reagent (Invitrogen, Carlsbad, USA) in serum-free medium according to the manufacturer’s protocol.

To obtain the stably transfected cell line, HCCC-9810 cells were transfected with MT1JP overexpression plasmid, and treated with 400 μg/ml G418 for 2 weeks. The single cells were selected out, and cultured without G418. After verification of MT1JP at RNA levels, the MT1JP-stably expressed cell lines were obtained.

### Cell counting kit-8 (CCK-8) assay

CCK-8 assay was performed to measure the cell viability. The cells were cultured in 96-well plates at 37 °C with 5% CO_2_. After culture for 0 h, 24 h, 48 h or 72 h, the cells were incubated with CCK-8 reagent (10 μl per well) (KeyGEN, Nanjing, China) for 2 h. The optical density of medium was detected with a microplate reader (BioTek, Winooski, VT, USA) at 450 nm.

### Flow cytometry

Flow cytometry was used for detection of apoptosis and cell cycle.

The cells were collected, and incubated with Annextin V-FITC reagent and propidium iodide at room temperature for 20 min in the dark. Then the cells were detected by flow cytometer (BD, Franklin Lakers, NJ, USA).

For cell cycle detection, the cells were collected and immobilized with 70% ethanol at 4 °C overnight. Then the cells were incubated with PI/RNaseA buffer at room temperature for 60 min in the dark, and used for detection by flow cytometer.

### Transwell assay

Transwell assay was used for detection of migration and invasion.

The cells were collected and counted. About 3 × 10^3^ cells were seeded into the upper chamber with serum-free medium, and the lower chamber was added with medium with 30% FBS. After culture for 24 h, the cells on the reverse surface of transwell membrane was fixed with 4% paraformaldehyde (Aladdin, Shanghai, China) and stained with 0.4% crystal violet (Amresco, Solon, OH, USA). The cells were photographed and counted under a microscope (XI53, Olympus, Tokyo, Japan) at 100× magnification.

For invasion analysis, the polycarbonate membrane of transwell chambers (Corning, NY, USA) was pre-coated with Matrigel at 37 °C. Approximately 1.5 × 10^4^ cells were seeded into upper chamber with serum-free medium, and medium with 30% FBS were added into the lower chamber. After culture for 24 h, the cells on the reverse surface was fixed and stained, and the cell number was counted under a microscope.

### Dual-luciferase assay

The binding between MT1JP and miR-18a-5p was analyzed by bioinformatic website RNAhybrid (https://bibiserv.cebitec.uni-bielefeld.de/rnahybrid/) [13]. The MT1JP sequence containing miR-18a-5p-bound region or mutant sequence was cloned into pmirGLO vector with *NheI* and *XhoI* sites, and cotransfected into 293 T cells with miR-18a-5p mimics. Twenty-four hours later, the cells were lysed, and the luciferase activity of pmirGLO vector was detected.

The candidate targets of miR-18a-5p were predicted by bioinformatic website targetscan (http://www.targetscan.org/vert_71/). The 3’UTR sequence of FBP1 containing miR-18a-5p-bound region or its mutant sequence was synthesized and inserted into pmirGLO vector with *NheI* and *XhoI* sites. 293 T cells were cotransfected with pmirGLO vector and miR-18a-5p mimics, and the activity of luciferase activity was determined.

### Immunofluorescent staining

The cells were pre-seeded on glass slides. After culture for certain times, the cells were fixed with 4% paraformaldehyde for 15 min, permeated with 0.1% TritonX-100 (Beyotime) for 30 min, and blocked with goat serum for 10 min at room temperature. Subsequently, the cells were incubated with antibody against FBP1 (1:100; cat. no. 12842–1-AP, Proteintech) or CK19 (1:50; cat. no. A0247, Abclonal) at 4 °C overnight. After washing with PBS, the cells were incubated with secondary antibody labeled with Cy3 (1:200; cat. no. A0516, Beyotime) at room temperature in the dark for 60 min, and counterstained with DAPI (Aladdin). Finally, the glass slide were mounted with anti-fading reagent (Solarbio, Beijing, China), and the cells were observed under a fluorescent microscope (BX53, Olympus) at 400× magnification.

### Xenograft model

Healthy BALB/c mice were purchased from HFK Biotechnology Co. Ltd. (Beijing, China), and kept in a controlled environment (12 h/12 h light/dark, 22 ± 1 °C) with free access to food and water. The animals were taken care of according to Guide for the Care and Use of Laboratory animal (8th edition, NIH), and the experimental procedures were approved by Ethics Committee of Affiliated Hospital of Qingdao University.

After accommodation for one week, the mice were subcutaneously injected with HCCC-9810 cells (5 × 10^5^ each mouse) with MT1JP stable overexpression or not (*n* = 6 in each group). One week later, the tumor size was measured every three days. Three weeks after injection, the mice were euthanized via overdose of pentobarbital sodium (200 mg/kg, intraperitoneal injection), and the tumors were isolated for detection.

### HE staining

The tumor isolated from mice were fixed in 4% paraformaldehyde overnight, and washed with flow water for 4 h. Then the tissue was dehydrated with ethanol of grading concentrations and xylene, embedded with paraffin, and cut into sections of 5 μm. The sections were deparaffinized with xylene and ethanol, and stained with hematoxylin (Solarbio). After soaking in 1% hydrochloric acid/ethanol for several seconds, the sections were stained with eosin (Sangon, Shanghai, China). Finally, the sections were dehydrated again, mounted with gum, and observed under a microscope (XI53, Olympus) at 200× magnification.

### TUNEL staining

The tumor tissue was made into paraffin sections as described previously. After deparaffinization, the sections were permeated with 0.1% Triton X-100, and blocked with 3% H_2_O_2_ at room temperature. Then the sections were incubated with TUNEL buffer (Roche, Basel, Switzerland) for 60 min at 37 °C in the dark, and incubated with Converter-POD reagent for 30 min at 37 °C. Subsequently, the sections were reacted with DAB substrate (Solarbio), and counterstained with hematoxylin. Finally, the sections were dehydrated, mounted and photographed with a microscope (BX53, Olympus) at 400× magnification.

### Immunohistochemical staining

The tumor tissues were used for immunohistochemical staining for detection of Ki-67 and FBP1. The tissues were made into sections as previously described. The sections were reacted with antigen repair buffer in boiling for 10 min, and blocked with 3% H_2_O_2_ and goat serum. Then the section were incubated with antibody against Ki-67 (1:100; cat. no. AF0198, Affinity) or FBP1 (1:100; cat. no. 12842–1-AP, Proteintech) at 4 °C overnight. After washing with PBS, the sections were incubated with secondary antibody labeled with HRP (1:500; Beyotime) at 37 °C for 60 min, and reacted with DAB substrate. After counterstaining with hematoxylin, the sections were dehydrated, mounted and observed with a microscope at (XI53, Olympus) 400× magnification.

### Statistical analysis

The data in this study were presented as meas±SD, and analyzed with GraphPad Prism 8.0. The data from two independent groups were analyzed with student’s t test. Comparisons among multiple groups were performed with one-way or two-way analysis of variance followed with Bonferroni post-hoc test. The correlation between MT1JP and clinical features were analyzed by Pearson χ^2^ test. The correlation between MT1JP and miR-18a-5p was analyzed with Pearson test. A *p* value less than 0.05 was considered as statistically significantly.

## Results

### MT1JP was downregulated in cholangiocarcinoma specimens

**Fig. 1:**
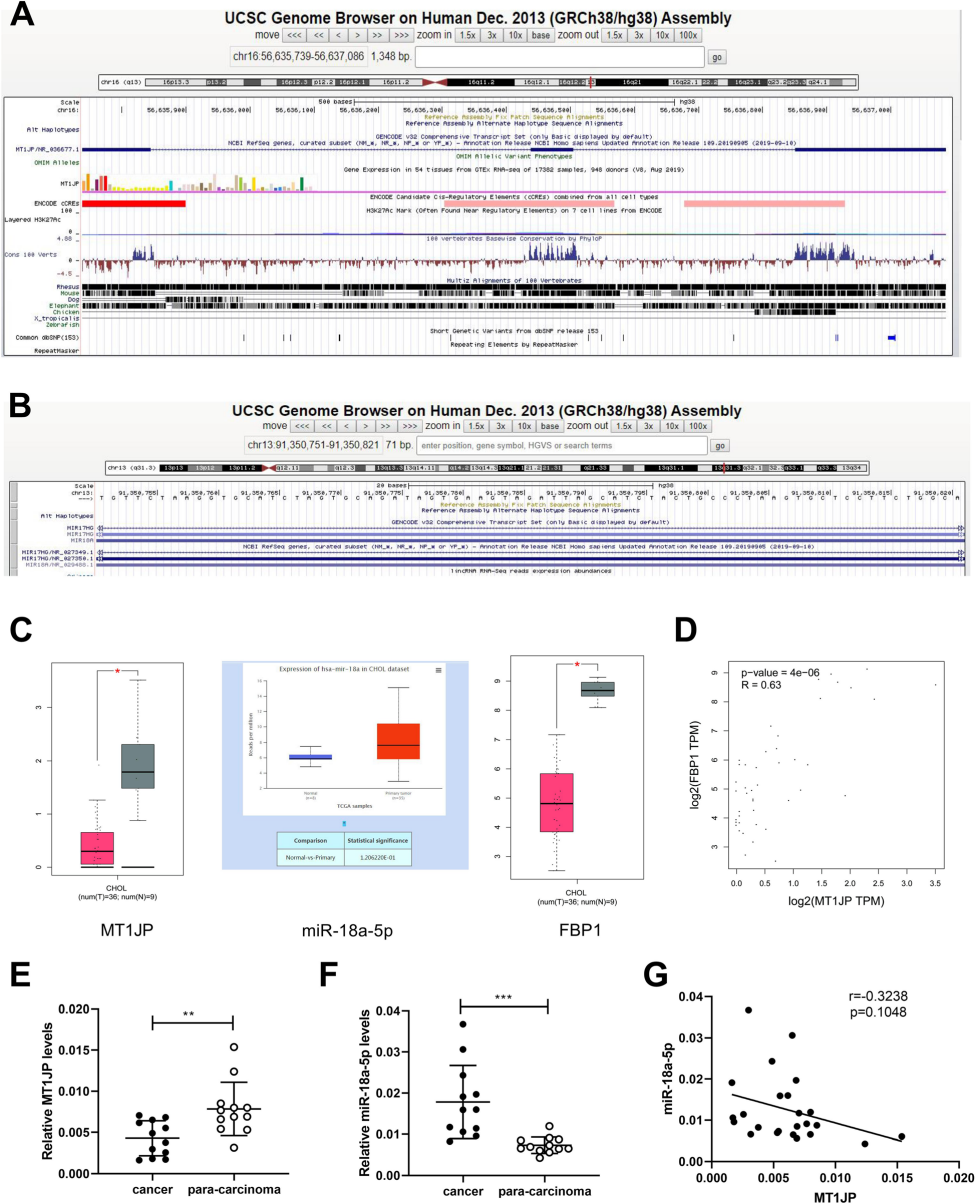
MT1JP was downregulated in cholangiocarcinoma specimens. A and B. The location of *MT1JP* and *miR-18a* genes from UCSC (https://genome.ucsc.edu/). C. The expression of MT1JP, miR-18a-5p and FBP1 in cholangiocarcinoma tissues from TCGA database. D. The correlation between MT1JP and FBP1 in cholangiocarcinoma tissues was analyzed by Pearson test in GEPIA website (http://gepia.cancer-pku.cn/). E. The expression of MT1JP was detected by real-time PCR in intrahepatic cholangiocarcinoma specimens and paired para-carcinoma tissues. F. The expression of miR-18a-5p was examined. G. The correlation between expression of MT1JP and miR-18a-5p in intrahepatic cholangiocarcinoma was analyzed with Pearson test

*MT1JP* gene is located in chr16:56635793–56,637,086, and *miR-18a* gene is located in chr13:91350751–91,350,721, as shown in Fig. 1a and b. According to TCGA database, MT1JP and FBP1 were downregulated and miR-18a-5p was upregulated in clinical cholangiocarcinoma tissues (Fig. 1c). In addition, GEPIA website showed that the expression level of MT1JP was positively correlated with that of FBP1 in cholangiocarcinoma tissues (Fig. 1d). Subsequently, twelve paired of intrahepatic cholangiocarcinoma specimens were used for detection of MT1JP and miR-18a-5p expression levels by real-time PCR. As shown in Fig. 1e and f, the MT1JP was decreased and miR-18a-5p was increased in intrahepatic cholangiocarnoma tissues, compared with para-carcinoma tissues, which was consistence with data from TCGA database. In addition, the correlation between MT1JP and miR-18a-5p was analyzed with pearson test, and Fig. 1g revealed that the expression of MT1JP was weakly negatively correlated with that of miR-18a-5p.

Next, the correlation between MT1JP expression and clinicopathological characteristics were analyzed. As shown in Table 2, the expression level of MT1JP was significantly related with TNM stage and lymph node metastasis.

**Table 2:** Correlation between MT1JP expression and intrahepatic cholangiocarcinoma patients’ clinicopathological characteristics

Variable	Category	No.of cases		χ2	***p*** value
		MT1JP high expression	MT1JP low expression		
		(***n*** = 15)	(n = 15)		
Age	< 60	5	8	1.22172	0.26902
	≥60	10	7		
Sex	Male	11	9	0.6	0.43858
	Female	4	6		
Tumor size	≤5 cm	14	10	3.33333	0.06789
	> 5 cm	1	5		
Histology differentiation	Well	0	1	1.03448	0.30911
	Moderate/Poor	15	14		
TNM stage	I-II	15	7	10.9091	0.00096***
	III-IV	0	8		
Lymph node metastasis	Present	1	10	11.6268	0.00065***
	Absent	14	5		
Distal metastasis	Present	0	1	1.03448	0.30911
	Absent	15	14		

The results in this section demonstrated that MT1JP was downregulated and miR-18a-5p was upregulated in cholangiocarcinoma specimens.

### MT1JP inhibited proliferation and promoted apoptosis in intrahepatic cholangiocarcinoma cells

**Fig. 2:**
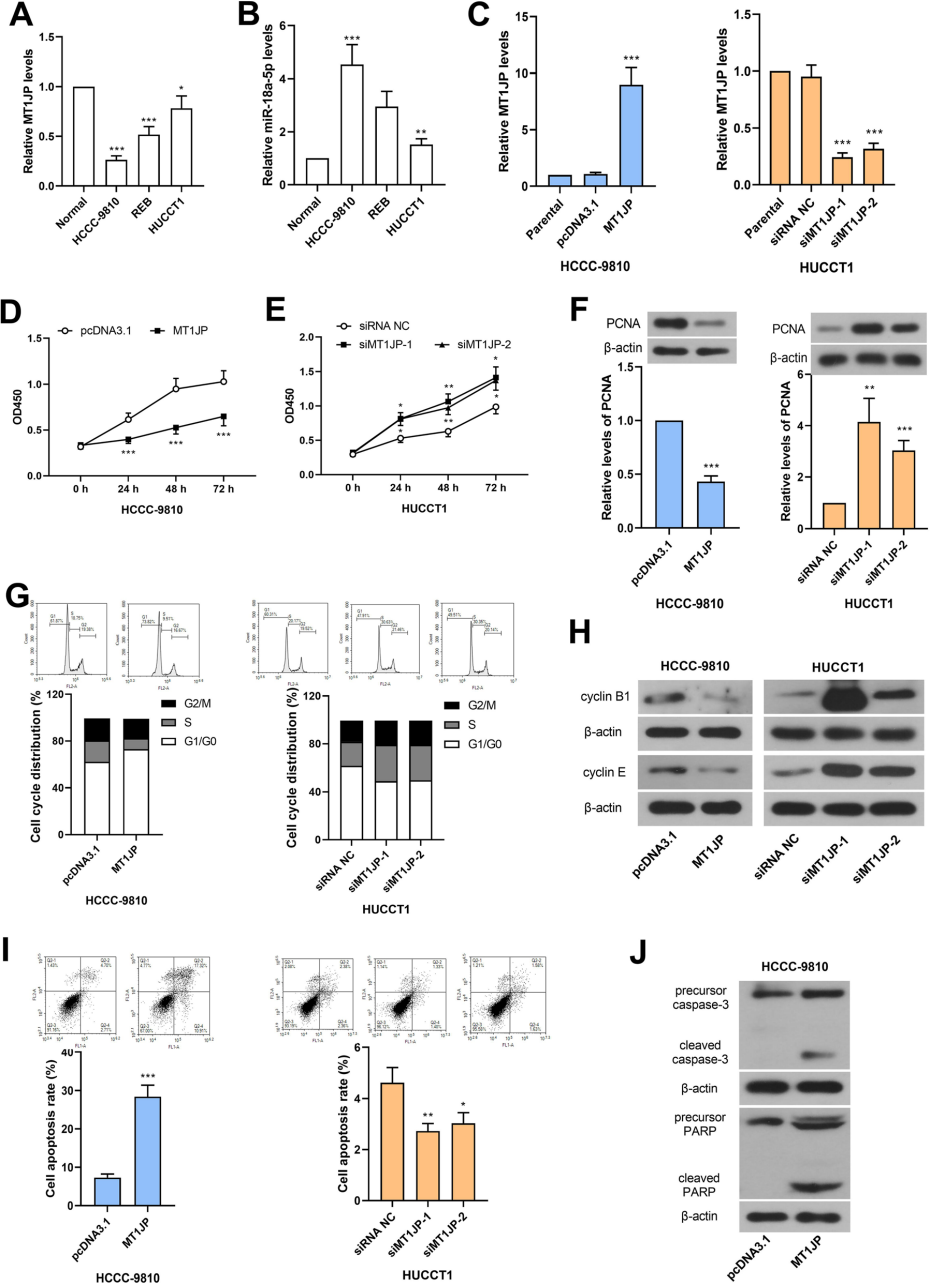
MT1JP inhibited proliferation and promoted apoptosis in intrahepatic cholangiocarcinoma cells. A and B. The levels of MT1JP and miR-18a-5p were detected by real-time PCR in normal primary intrahepatic cholangetic epithelial cells and several intrahepatic cholangiocarcinoma cell lines. C. The MT1JP levels were verified in HCCC-9810 and HUCCT1 cells after overexpression or knockdown. D and E. CCK-8 assay was used for detection of cell viability. F. The PCNA expression levels were detected by western blot after ectopic expression or silencing of MT1JP. (The western blot bands were cropped from Fig. S1 and S2.) G. Flow cytometry was used for cell distribution in each phase. H. The expression levels of several cell cycle proteins were detected. (The western blot bands were cropped from Fig. S3, S4, S5 and S6.) I. Flow cytometry was performed for detection of cell apoptosis. J. The expression levels of several apoptosis proteins were examined after MT1JP overexpression in HCCC-9810 cells. (The western blot bands were cropped from Fig. S7 and S8.) (*p < 0.05, **p < 0.01, ***p < 0.001, compared with normal, pcDNA3.1 or siRNA NC; the original images of western blot were shown in supplementary Fig. 1–8)

As MT1JP was decreased in intrahepatic cholangiocarcinoma tissues, its expression was examined in several intrahepatic cholangiocarcinoma cell lines and normal primary intrahepatic cholangetic epithelial cells. As shown in Fig. 2a and b, MT1JP was decreased and miR-18a-5p was increased in intrahepatic cholangiocarcinoma cells. Among these tumor cell lines, MT1JP was lowest in HCCC-9810 cells and highest in HUCCT1 cells, which was opposite with that of miR-18a-5p (Fig. 2a and b). In order to investigate the roles of MT1JP in cholangiocarcinoma cells, MT1JP overexpresion plasmid was transfected into HCCC-9810 cells, and its siRNA was transfected into HUCCT1 cells. The effectiveness of overexpression and silencing was confirmed by real-time PCR (Fig. 2c).

Next, the proliferation and apoptosis was detected. CCK-8 assay showed that MT1JP inhibited cell viability in cholangiocarcinoma cells (Fig. 2d and e). The expression level of PCNA was decreased after MT1JP overexpression and increased after MT1JP knockdown (Fig. 2f). The flow cytometry results revealed that MT1JP delayed G1/S and S/G2 transition in HCCC-9810 cells, and the silencing of MT1JP accelerated cell cycle transition in HUCCT1 cells (Fig. 2g), which was supported by expression of cyclin B1 and cyclin E (Fig. 2h). In addition, the ectopic expression of MT1JP enhanced apoptosis in HCCC-9810 cells, evidenced by expression changes of cleaved caspase-3 and cleaved PARP (Fig. 2i and j). The knockdown slightly inhibited the apoptosis in HUCCT1 cells (Fig. 2i).

The results in this sections suggested that MT1JP inhibited proliferation and promoted apoptosis in intrahepatic cholangiocarcinoma cells.

### MT1JP suppressed migration and invasion in intrahepatic cholangiocarcinoma cells

**Fig. 3:**
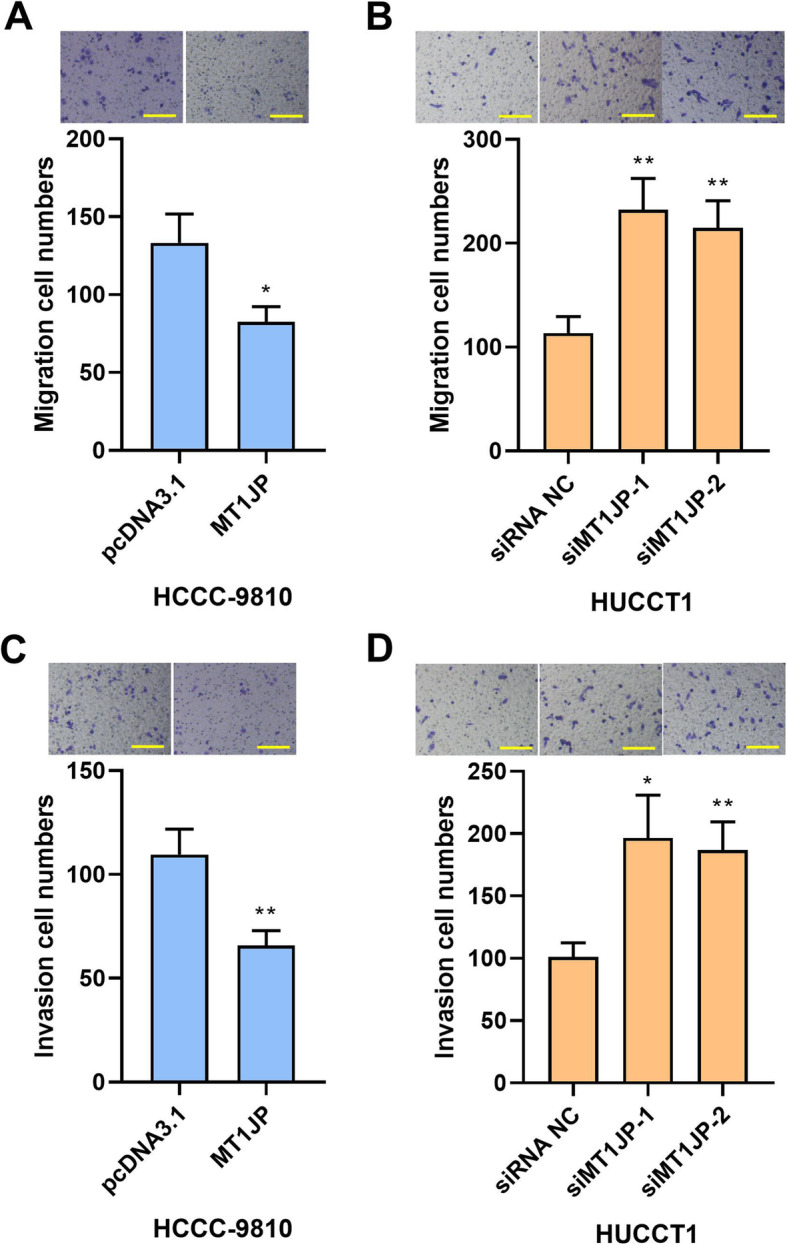
MT1JP suppressed migration and invasion in intrahepatic cholangiocarcinoma cells. A and B. The migratory ability of HCCC-9810 (A) and HUCCT1 (B) after MT1JP overexpression or knockdown was determined by transwell assay. C and D. Transwell assay supplemented with Matrigel was performed for detection of invasion of intrahepatic cholangiocarcinoma cells. (the scale bar represented as 200 μm) (*p < 0.05, **p < 0.01, compared with pcDNA3.1 or siRNA NC)

Next, the migration and invasion ability of intrahepatic cholangiocarcinoma cells were determined by transwell assay with or without Matrigel. As shown in Fig. 3, the overexpression of MT1JP suppressed migration and invasion in HCCC-9810 cells, and the silencing of MT1JP enhanced migration and invasion in HUCCT1 cells.

The results in this section suggested that MT1JP suppressed migration and invasion in intrahepatic cholangiocarcinoma cells.

### MT1JP bound to miR-18a-5p as a sponge and regulated the expression of FBP1

**Fig. 4:**
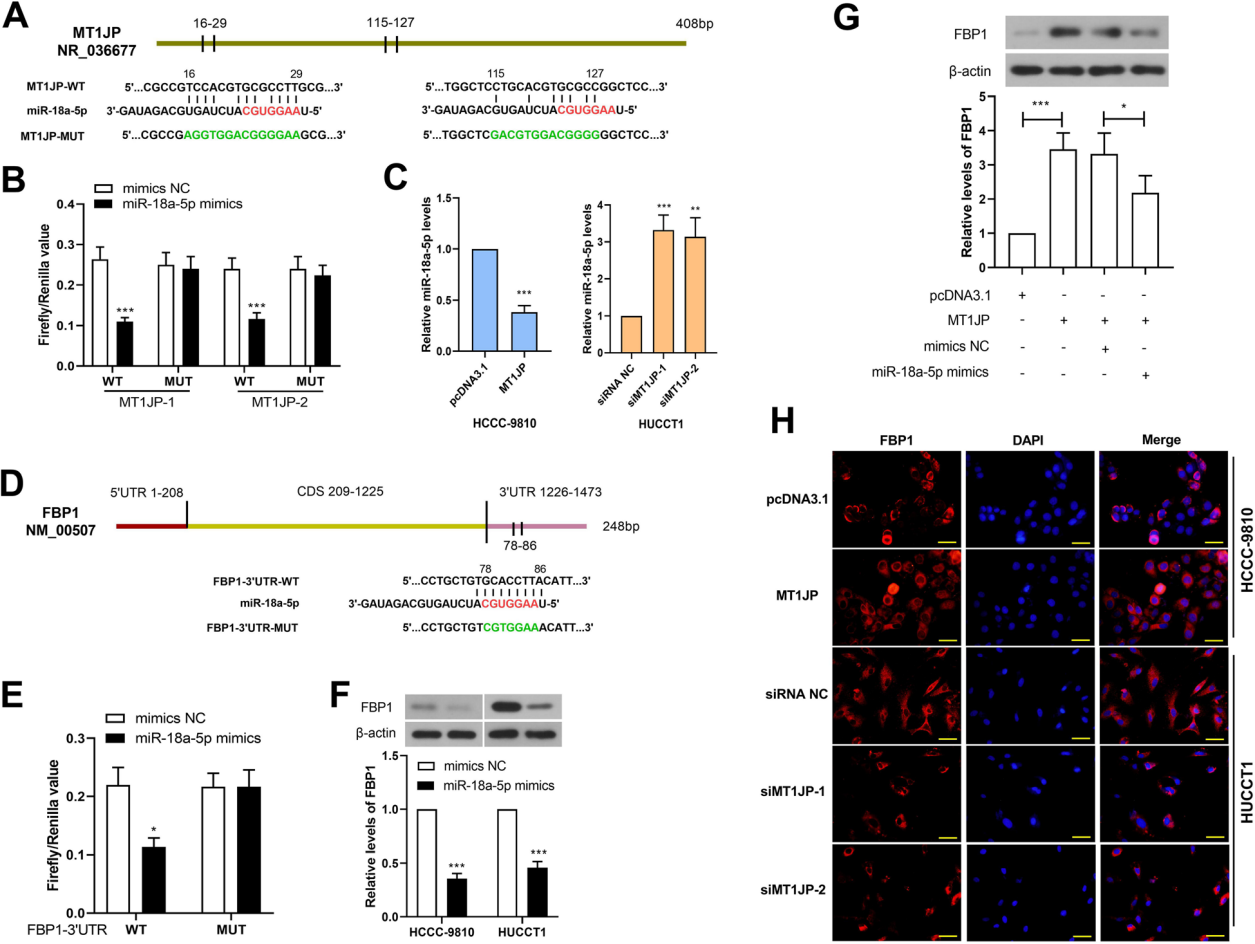
MT1JP bound to miR-18a-5p as a sponge and regulated the expression of FBP1. A. The sequence of MT1JP was bound by miR-18a-5p at 16–29 bp and 115–127 bp (miR-18a-5p seed sequences were shown in red, and the mutant sequences were shown in green). B. Dual-luciferase assay was performed to verify the binding of miR-18a-5p to MT1JP. C. The expression level of miR-18a-5p was detected by real-time PCR after overexpression or knockdown of MT1JP. D. The 3’UTR of FBP1 was bound by miR-18a-5p at 78–86 bp (miR-18a-5p seed sequences were shown in red, and the mutant sequences were shown in green). E. Dual-luciferase assay was carried out to confirm the binding of miR-18a-5p to FBP1 3’UTR. F. The expression level of FBP1 was determined in HCCC-9810 and HUCCT1 cells after transfection of miR-18a-5p mimics. (The western blot bands were cropped from Fig. S9 and S10.) G. The expression level of FBP1 after ectopic expression of MT1JP or/and miR-18a-5p. (The western blot bands were cropped from Fig. S11.) H. Immunofluorescent staining was used to detect the expression and distribution of FBP1 after ectopic expression or silencing of MT1JP. (the scale bar represented as 50 μm) (*p < 0.05, **p < 0.01, ***p < 0.001, compared with pcDNA3.1, siRNA NC, or mimics NC)

As the negative correlation between MT1JP and miR-18a-5p expression levels and the predicted positive correlation between MT1JP and FBP1, the relationship among MT1JP, miR-18a-5p FBP1 was detected. The bioinformatic website RNAhybrid predicted two miR-18a-5p-binding sites in MT1JP sequence (Fig. 4a), and FBP1 was predicted to be bound by miR-18a-5p in its 3’UTR sequence (Fig. 4d). Dual-luciferase reporter assay revealed that miR-18a-5p reduced the luciferase activity of dual-luciferase reporter containing MT1JP site-1 and site-2, not their mutant sites (Fig. 4b), and MT1JP negatively regulated the expression of miR-18a-5p (Fig. 4c). In addition, the binding of miR-18a-5p to FBP1 3’UTR was verified by dual-luciferase reporter assay (Fig. 4e), and western blot demonstrated that the expression levels of FBP1 was declined after transfection of miR-18a-5p mimics (Fig. 4f). Therefore, we hypothesized that MT1JP regulated expression of FBP1 by sponging miR-18a-5p. To confirm the hypothesis, the expression of FBP1 was examined after overexpression of MT1JP and miR-18a-5p. As shown in Fig. 4g, the expression of FBP1 was increased after ectopic expression of MT1JP, which was reversed after transfection of miR-18a-5p mimics, suggested that MT1JP competitively bound to miR-18a-5p with FBP1. The immunofluorescent staining confirmed that MT1JP promoted the expression of FBP1 (Fig. 4h).

The results in this section suggested that MT1JP facilitated FBP1 expression via acting as a sponge to bind miR-18a-5p.

### The effect of MT1JP was attenuated by overexpression of miR-18a-5p or knockdown of FBP1

**Fig. 5:**
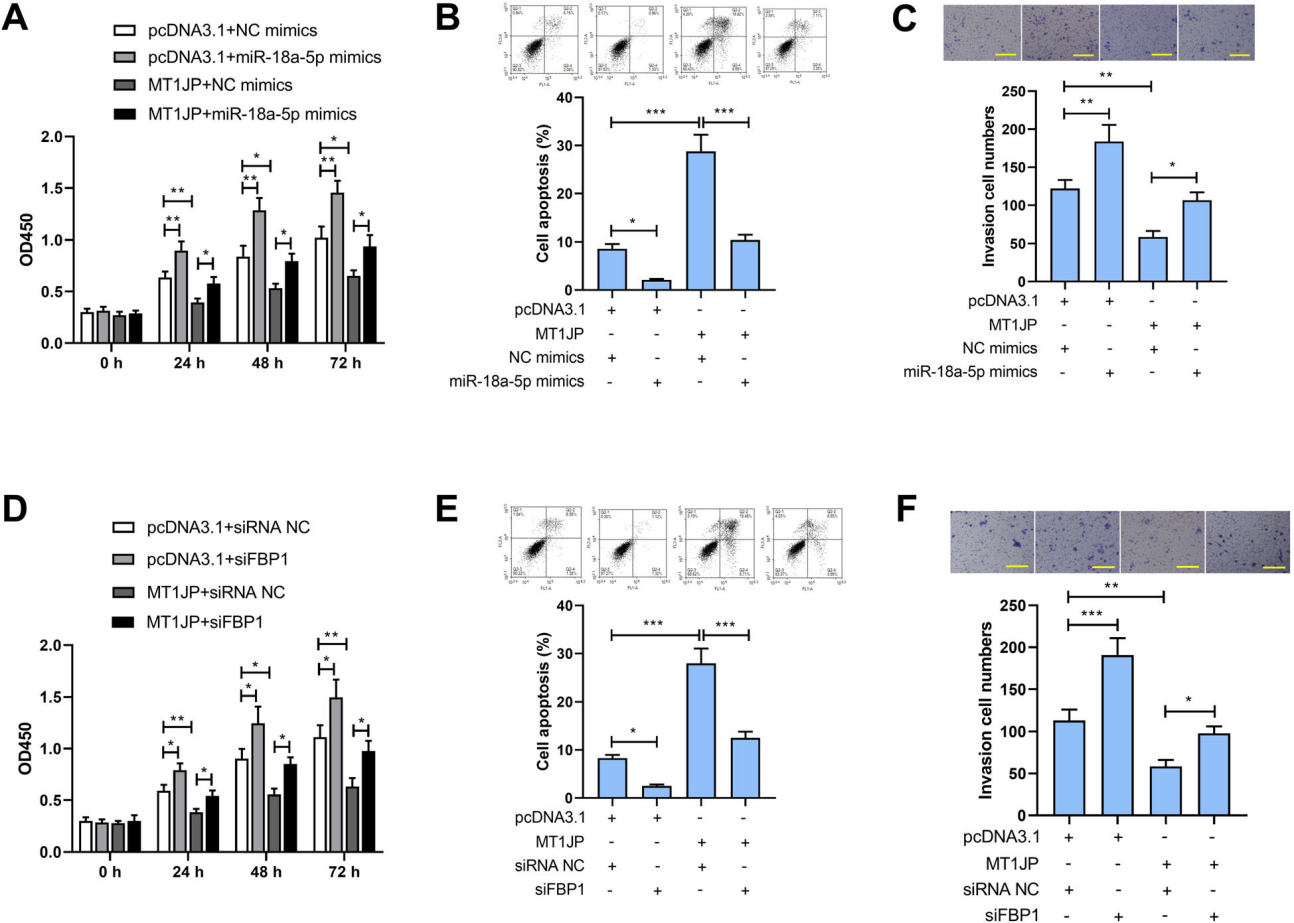
The effect of MT1JP was attenuated by overexpression of miR-18a-5p or knockdown of FBP1. A. CCK-8 assay was used to measure the cell ability in HCCC-9810 cells after transfection of MT1JP and miR-18a-5p mimics. B. Flow cytometry was used for measurement of apoptosis. C. Transwell assay supplemented with Matrigel was used for cell invasive ability detection. D. The cell viability was detected by CCK-8 assay in HCCC-9810 cells after overexpression of MT1JP and knockdown of FBP1. E. The cell apoptosis was detected by flow cytometry in HCCC-9810 cells. F. The invasive ability was determined by transwell assay supplemented with Matrigel. (the scale bar represented as 200 μm) (*p < 0.05, **p < 0.01, ***p < 0.001)

In order to further investigate the mechanism of MT1JP function, the MT1JP overexpression plasmid was cotransfected with miR-18a-5p or FBP1 siRNA. As shown in Fig. 5, increase of cell viability and invasive ability, and the decrease of apoptosis of induced by MT1JP were abolished by transfection of miR-18a-5p mimics in HCCC-9810 cells (Fig. 5a-c). Similarly, the effect of MT1JP on cell viability, apoptosis and invasion in HCCC-9810 cells were attenuated by FBP1 siRNA (Fig. 5d-f). These results demonstrated that MT1JP played its role by binding to miR-18a-5p and facilitated the expression of FBP1.

### MT1JP restrained tumorigenesis in nude mice

**Fig. 6:**
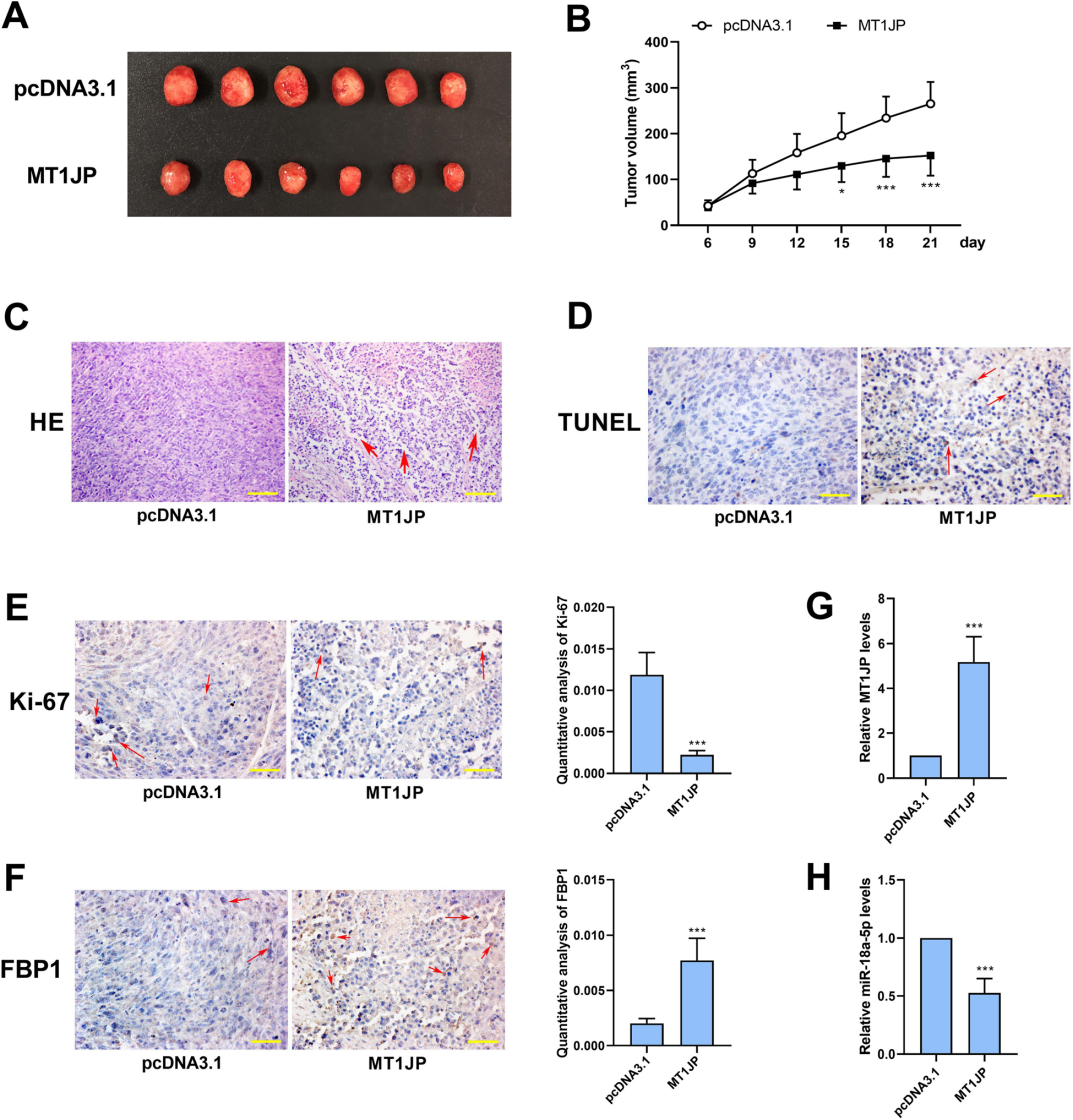
MT1JP restrained tumorigenesis in nude mice. HCCC-9810 cells stably transfected with MT1JP or control vector were subcutaneously injected into nude mice, and the tumors were isolated after three weeks. A. The subcutaneous tumors. B. The tumor volume. C. HE staining was performed to detect the pathological changes of tumors. (the scale bar represented as 100 μm, the arrows indicated intervals caused by cell death) D. TUNEL assay was used to detect cell apoptosis in tumors. E. Immunohistochemical staining was used to detect the expression of Ki-67. F. Immunohistochemical staining was used to detect the expression of FBP1. (the scale bar in D-F represented as 50 μm, and arrows in D-F indicated positive cells) G and H. Real-time PCR was performed for measurement of MT1JP and miR-18a-5p expression in tumors. (*n* = 6, *p < 0.05, ***p < 0.001, compared with pcDNA3.1)

To investigate the effect of MT1JP on tumorigenesis of intrahepatic cholangiocarcinoma cells, the HCCC-9810 cells with MT1JP stable expression were subcutaneously injected into nude mice (*n* = 6). The tumors were isolated three weeks after injection. Figure 6a and b showed that MT1JP significantly inhibited tumorigenesis in nude mice. HE staining, TUNEL and immunohistochemical staining of Ki-67 revealed that MT1JP led to cell necrosis and apoptosis, and suppressed cell proliferation in tumors (Fig. 6c-e). The real-time PCR and immunohistochemical staining results demonstrated that in MT1JP-overexpressed tumors, the expression of miR-18a-5p was declined, and that of FBP1 was elevated (Fig. 6f-g), which supported the hypothesis that MT1JP bound to miR-18a-5p and facilitated the expression of FBP1.

The results in this section suggested that MT1JP retarded tumorigenesis of intrahepatic cholangiocarcinoma cells in nude mice.

## Discussion

In this study, we found that MT1JP was downregulated in clinical cholangiocarcinoma specimens, and its expression was correlated with TNM stage and lymph node metastasis. Gain- and loss-of-function experiments demonstrated that MT1JP inhibited cell proliferation, cell cycle transition, migration and invasion, and promoted apoptosis in cholangiocarcinoma cells. Xenograft experiments showed that MT1JP suppressed tumorigenesis in nude mice. In addition, miR-18a-5p was increased in cholangiocarcinoma tissues, which was negatively correlated with that of MT1JP. MT1JP bound to miR-18a-5p as a sponge, and enhanced the expression of a target of miR-18a-5p, FBP1.

The tumor-suppressing role of MT1JP has been reported in multiple cancer cells, including breast cancer, glioblastoma, bladder cancer and gastric cancer [11, 14–16]. The expression of lncRNA is often tissue-specific. We first demonstrated its effect on cholangiocarcinoma cells. The *MT1JP* gene locates in chromosome 16 in a cluster consisting of several homologous protein-coding genes of the metallothionein family [10]. MT1JP has been known to bind to miRNAs to play its roles. In this study, we demonstrated that MT1JP bound to miR-18a-5p to facilitate the expression of FBP1. MiR-18a-5p belongs to miR-17-92 cluster, plays tumor-promoting roles in colorectal cancer, lung cancer and renal cell carcinoma cells [17–19]. However, one study reported that miR-18a-5p was downregulated in breast cancer tissues, and inhibited migration and invasion in breast cancer cells [20]. These reporters suggested that the role of miR-18a-5p may be different in various cancers. In our study, miR-18a-5p was increased in cholangiocarcinoma specimens, and its overexpression enhanced proliferation and invasion, and reduced apoptosis in cholangiocarcinoma cells. FBP1 was confirmed as a target of miR-18a-5p. FBP1 is a gluconeogenesis regulatory enzyme, and catalyzes the hydrolysis of fructose-1,6-bisphosphate to fructose-6-phosphate and inorganic phosphate [21]. In our previous study, FBP1 was demonstrated to inhibit proliferation and metastasis in cholangiocarcinoma cells by regulating Wnt/β-catenin signaling pathway [12]. MT1JP may function via miR-18a-5p/FBP1/Wnt/β-catenin axis.

In this study, we analyzed the correlation between MT1JP expression and clinical characteristics of thirty intrahepatic cholangiocarcinoma patients, and found that the expression level of MT1JP was related with TNM stage and lymph node metastasis. The MT1JP low expression is more common in patients with advanced stage or/and existence of lymph node metastasis. Moreover, the expression of MT1JP was significantly decreased in intrahepatic cholangiocarcinoma samples. These finding suggested that MT1JP may participate in proliferation and metastasis of intrahepatic cholangiocarcinoma cells. Unlimited growth and metastasis are most important and terrible characteristics of malignant tumors. The malignant behaviors of cancer cells contain excessive proliferation, reduced apoptosis, uncontrolled migratory and invasive abilities and so on. The three cell lines, HCCC-9810, RBE and HUCCT1, are isolated from intrahepatic cholangiocarcinoma tissues, and present as epithelial cells [22–24]. Overexpression of MT1JP inhibited proliferation and cell cycle transition, and induced apoptosis in HCCC-9810 cells, evidenced by the decreased expression level of PCNA, cyclin B1, cyclin E, and increased expression level of cleaved caspase-3 and PARP. Migratory and invasive abilities were also reduced after ectopic expression of MT1JP. Opposite results were observed in MT1JP-silenced HUCCT1 cells. These results were consistent with previous papers about MT1JP in other cancers.

**Fig. 7:**
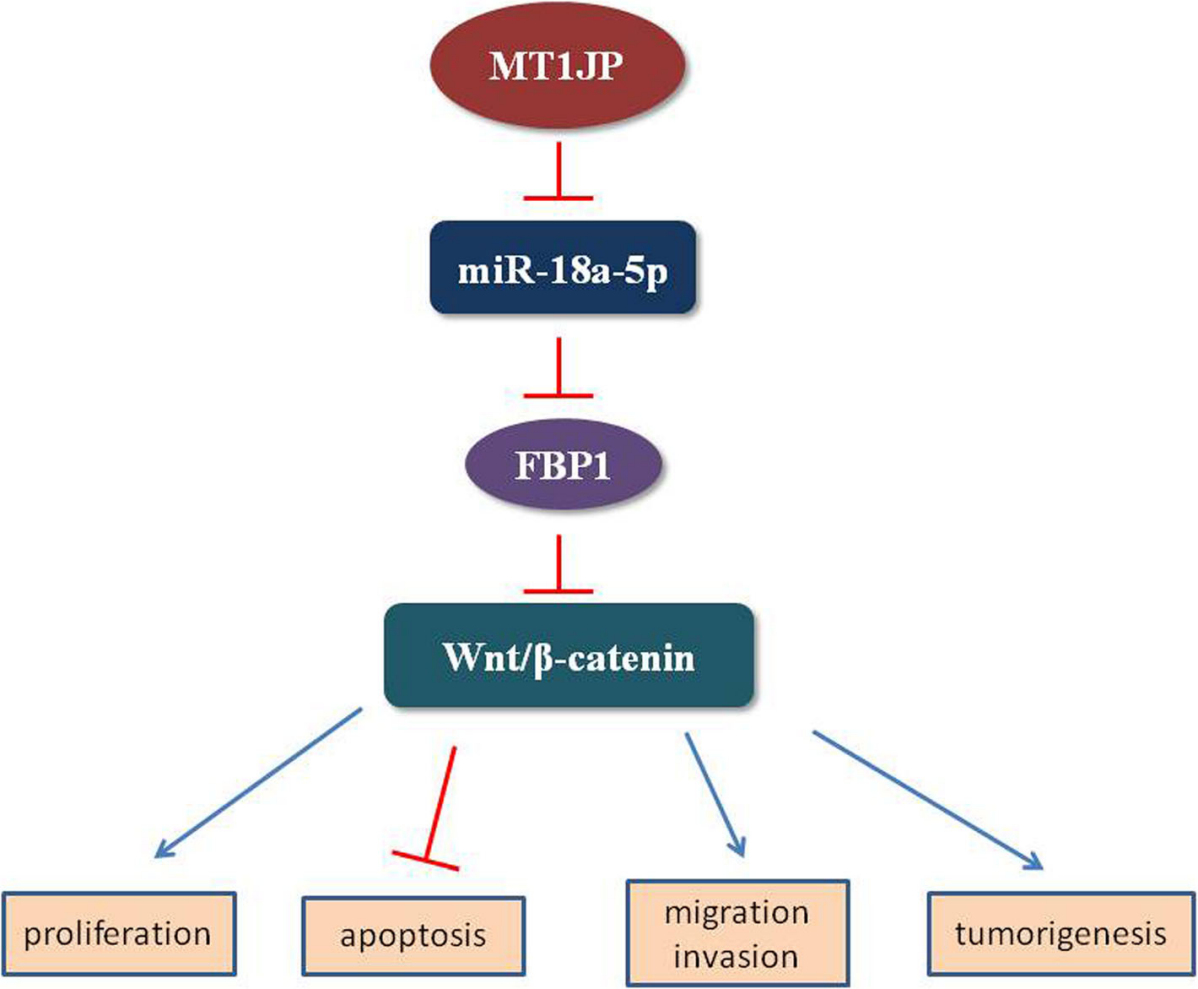
MT1JP regulated malignancies in intrahepatic cholangiocarcinoma cells via miR-18a-5p/FBP1/Wnt/β-catenin axis

We came up with a hypothesis that MT1JP acted as a miRNA sponge, and bound to miR-18a-5p. In our results, the miR-18a-5p level was inhibited by MT1JP, suggested that MT1JP may caused miRNA degradation. The expression of FBP1 was inhibited by miR-18a-5p, but enhanced by MT1JP, suggested that MT1JP competed with FBP1 for binding to miR-18a-5p. Previously, we demonstrated that FBP1 inhibited malignancies by inactivating Wnt/β-catenin signaling. So we hypothesized that MT1JP may function via miR-18a-5p/FBP1/Wnt/β-catenin axis (Fig. 7). This hypothesis needs to be supported by more experiment results.

## Conclusions

In this study, we demonstrated that lncRNA MT1JP was downregulated in intrahepatic cholangiocarcinoma tissues. MT1JP inhibited proliferation, migration, invasion and tumorigenesis and enhanced apoptosis in intrahepatic cholangiocarcinoma cells as a miRNA sponge to bind to miR-18a-5p to facilitate the expression of FBP1. These findings may provide novel diagnostic and therapeutic targets.

## Supplementary Information

**Additional file 1 Fig. S1** The western blot bands of PCNA and the internal control after MT1JP overexpression in HCCC-9810 cells.

**Additional file 2 Fig. S2** The western blot bands of PCNA and the internal control after silencing of MT1JP in HUCCT1 cells.

**Additional file 3 Fig. S3** The western blot bands of cyclin B1 and the internal control after MT1JP ectopic expression in HCCC-9810 cells.

**Additional file 4 Fig. S4** The western blot bands of cyclin E and the internal control after MT1JP overexpression in HCCC-9810 cells.

**Additional file 5 Fig. S5** The western blot bands of cyclin B1 and the internal control after MT1JP knockdown in HUCCT1 cells.

**Additional file 6 Fig. S6** The western blot bands of cyclin E and the internal control after interference of MT1JP in HUCCT1 cells.

**Additional file 7 Fig. S7** The western blot bands of caspase-3 precursor, cleaved caspase-3 and the internal control after MT1JP overexpression in HCCC-9810 cells.

**Additional file 8 Fig. S8** The western blot bands of PARP precursor, cleaved PARP and the internal control after ectopic expression of MT1JP in HUCCT1 cells.

**Additional file 9 Fig. S9** The western blot bands of FBP1 and the internal control after transfection of miR-18a-5p mimics in HCCC-9810 cells.

**Additional file 10 Fig. S10** The western blot bands of FBP1 and the internal control after overexpression of miR-18a-5p in HUCCT1 cells.

**Additional file 11 Fig. S11** The western blot bands of FBP1and the internal control after overexpression of MT1JP or/and miR-18a-5p.

**Additional file 12 Fig. S12**. Immunofluorescent staining of CK19 was used for identification of human primary intrahepatic cholangetic epithelial cells.

## Data Availability

The data in this study will not be shared due to the policy of the Ethic Committee.

## Abbreviations

MT1JP: metallothionein 1 J pseudogene
FBP1: fructose-1,6-bisphosphatase 1.
lncRNA: long noncoding RNA.
PCNA: anti-proliferating cell nuclear antigen.
PARP: anti-poly ADP-ribose polymerase.
TUNEL: terminal deoxynucleoitidyl transferase mediated nick end labeling.
TNM stage: tumor-node-metastasis stage.
3’UTR: 3’untranslated region.
