# Supplementary figures and images for: LncRNA MT1JP plays a protective role in intrahepatic cholangiocarcinoma by regulating miR-18a-5p/FBP1 axis
Source: BMC Cancer. 2021 Feb 8;21:142. doi: 10.1186/s12885-021-07838-0 (PMC7871555; doi:10.1186/s12885-021-07838-0)

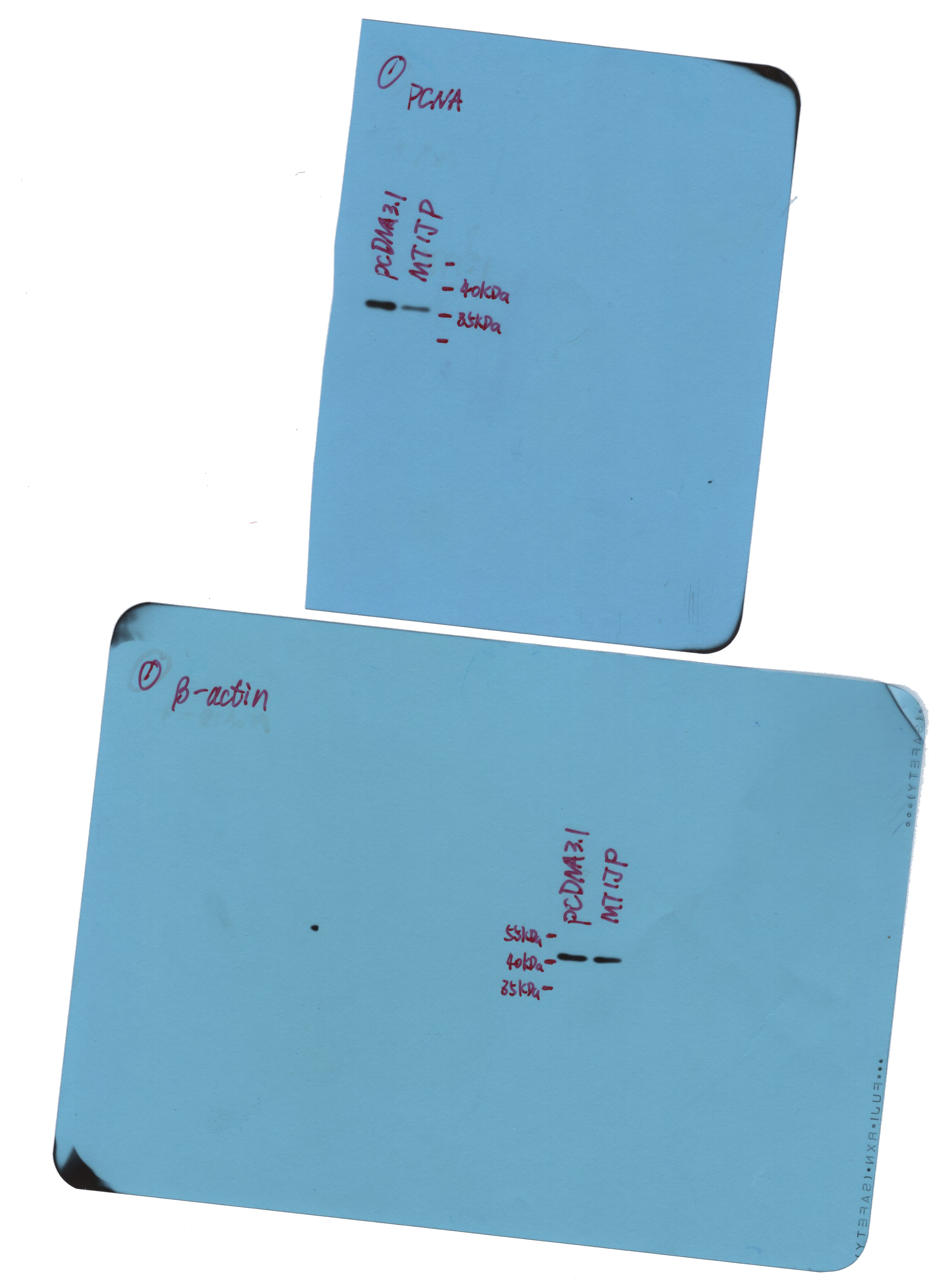

Supplement: Supplementary file 1 — Additional file 1 Fig. S1 The western blot bands of PCNA and the internal control after MT1JP overexpression in HCCC-9810 cells. [file 12885_2021_7838_MOESM1_ESM.jpg]

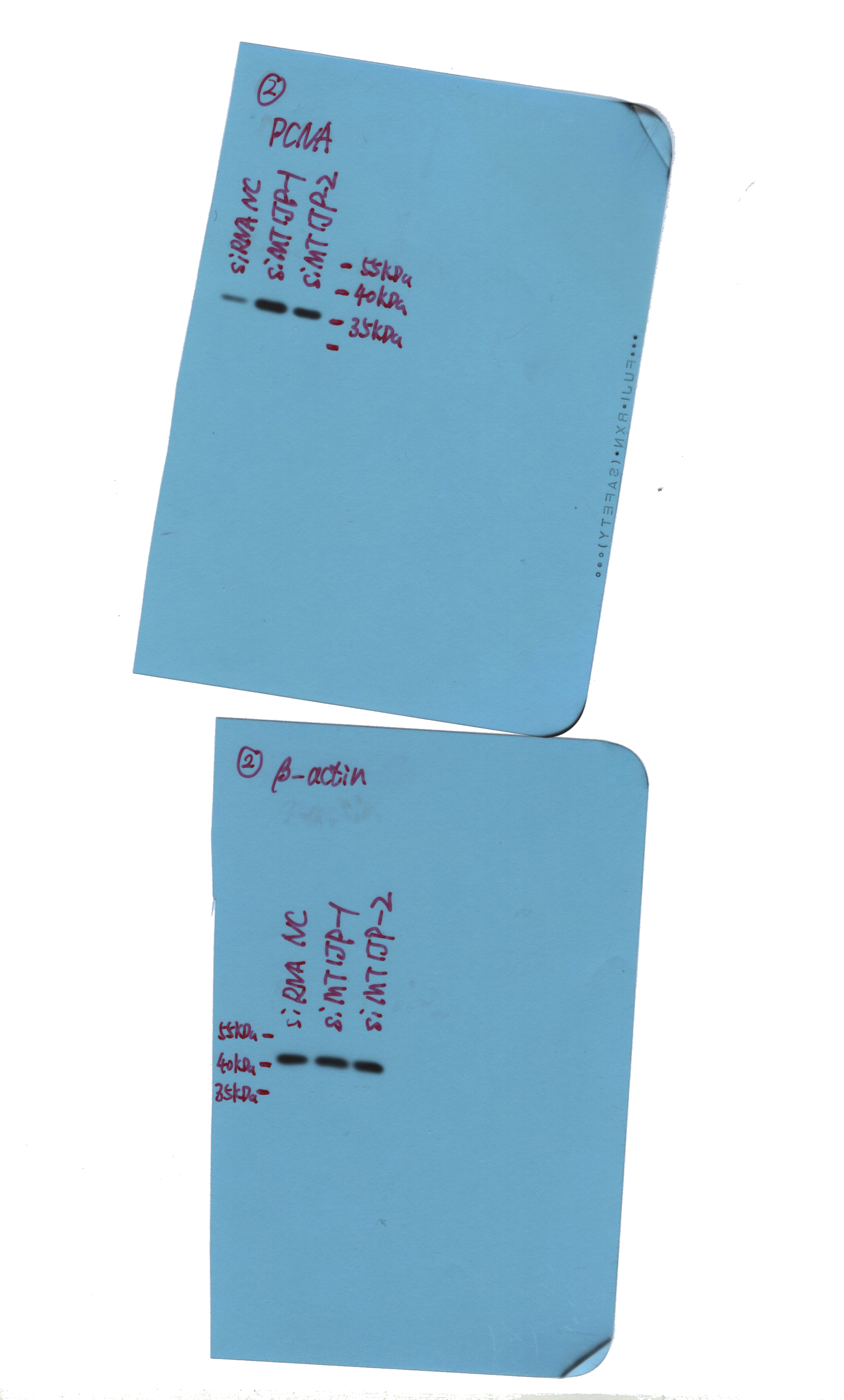

Supplement: Supplementary file 2 — Additional file 2 Fig. S2 The western blot bands of PCNA and the internal control after silencing of MT1JP in HUCCT1 cells. [file 12885_2021_7838_MOESM2_ESM.jpg]

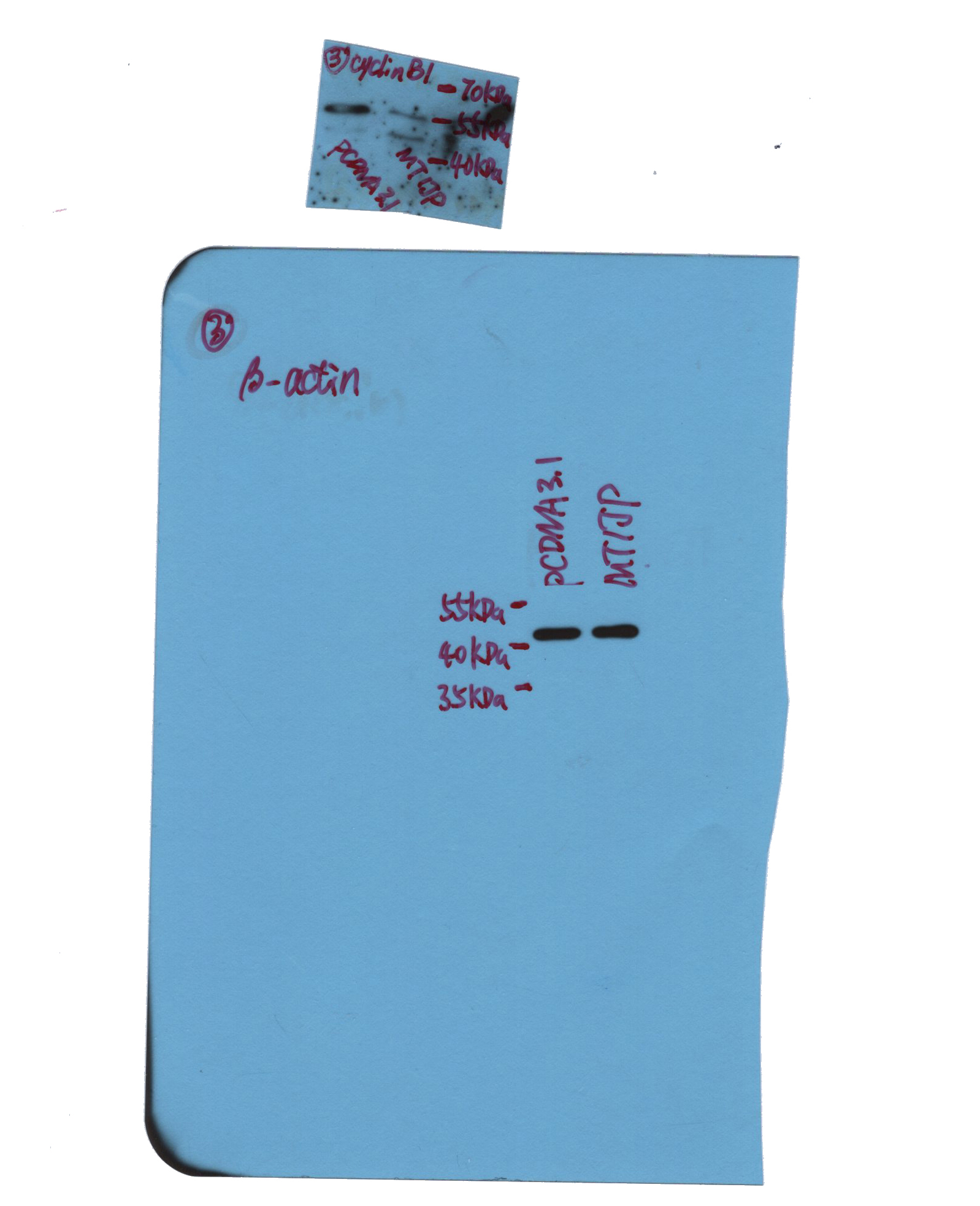

Supplement: Supplementary file 3 — Additional file 3 Fig. S3 The western blot bands of cyclin B1 and the internal control after MT1JP ectopic expression in HCCC-9810 cells. [file 12885_2021_7838_MOESM3_ESM.jpg]

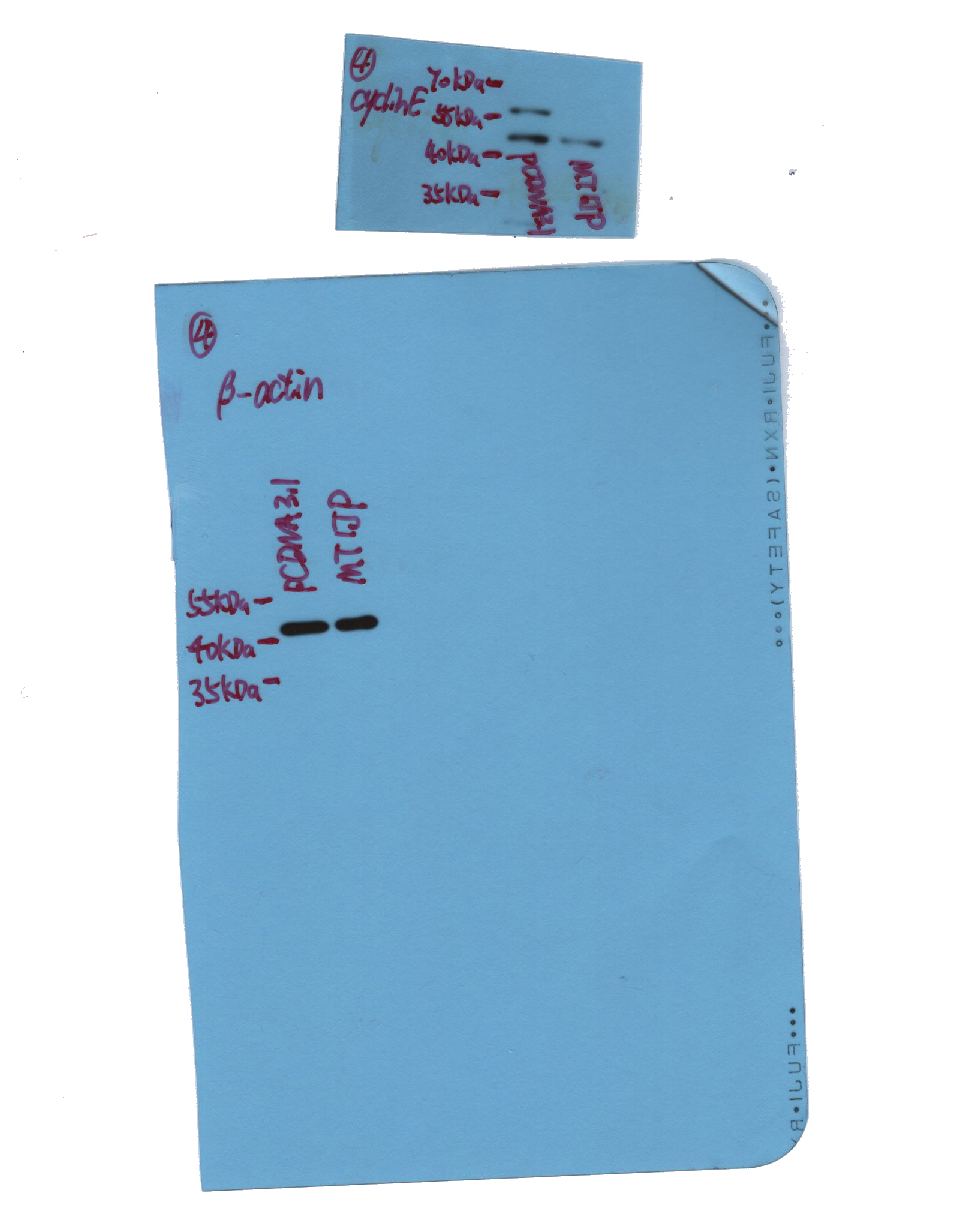

Supplement: Supplementary file 4 — Additional file 4 Fig. S4 The western blot bands of cyclin E and the internal control after MT1JP overexpression in HCCC-9810 cells. [file 12885_2021_7838_MOESM4_ESM.jpg]

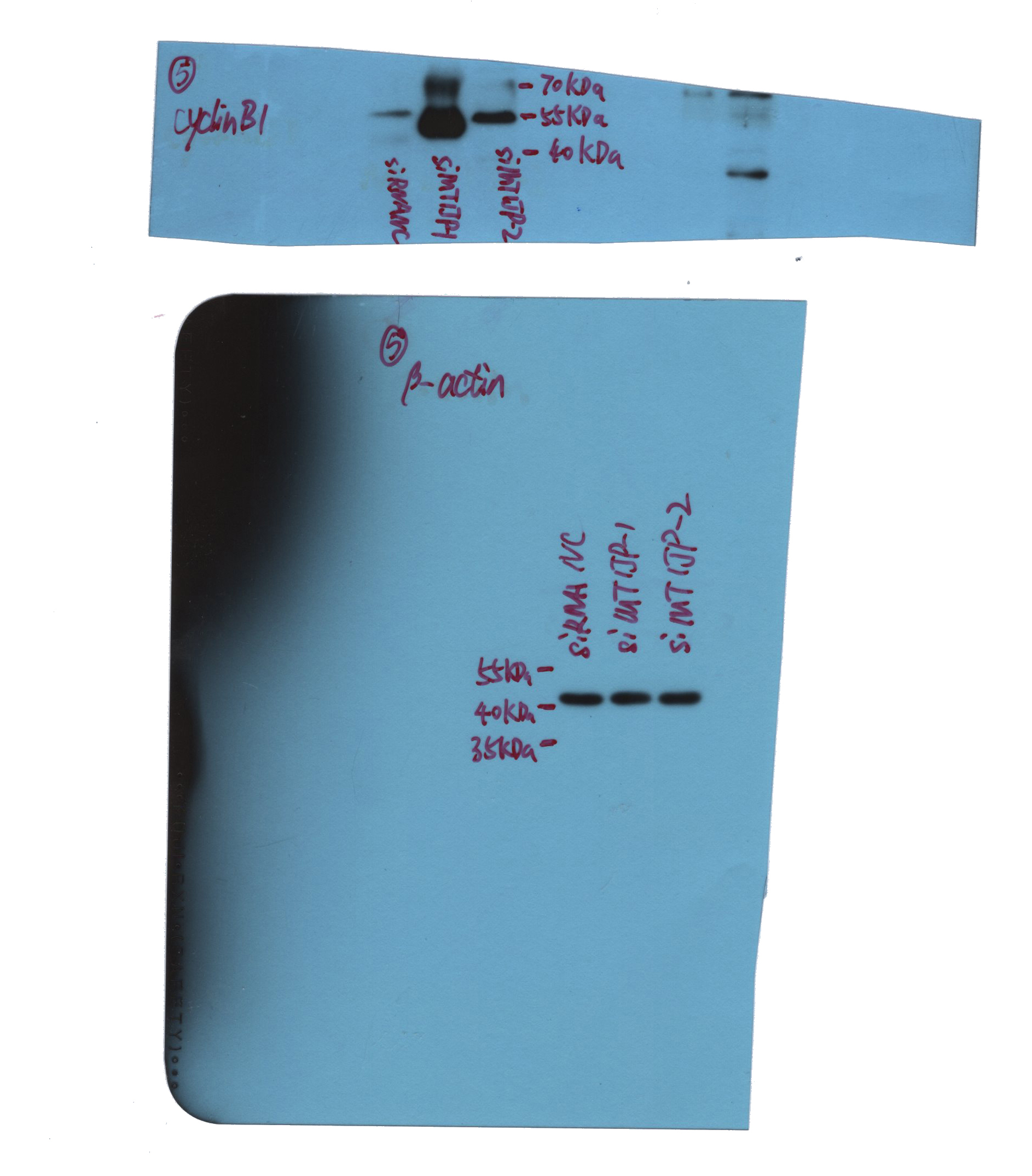

Supplement: Supplementary file 5 — Additional file 5 Fig. S5 The western blot bands of cyclin B1 and the internal control after MT1JP knockdown in HUCCT1 cells. [file 12885_2021_7838_MOESM5_ESM.jpg]

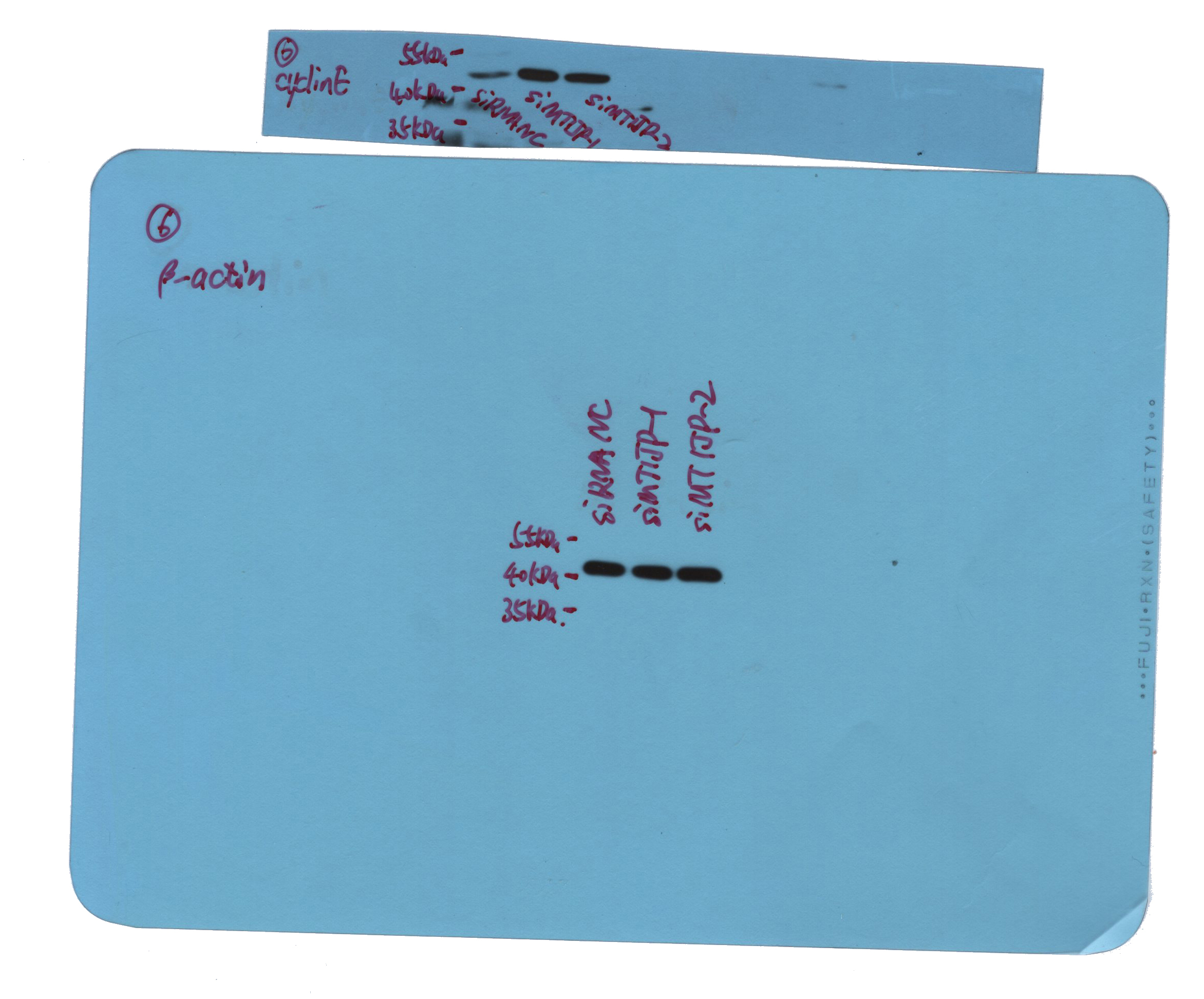

Supplement: Supplementary file 6 — Additional file 6 Fig. S6 The western blot bands of cyclin E and the internal control after interference of MT1JP in HUCCT1 cells. [file 12885_2021_7838_MOESM6_ESM.jpg]

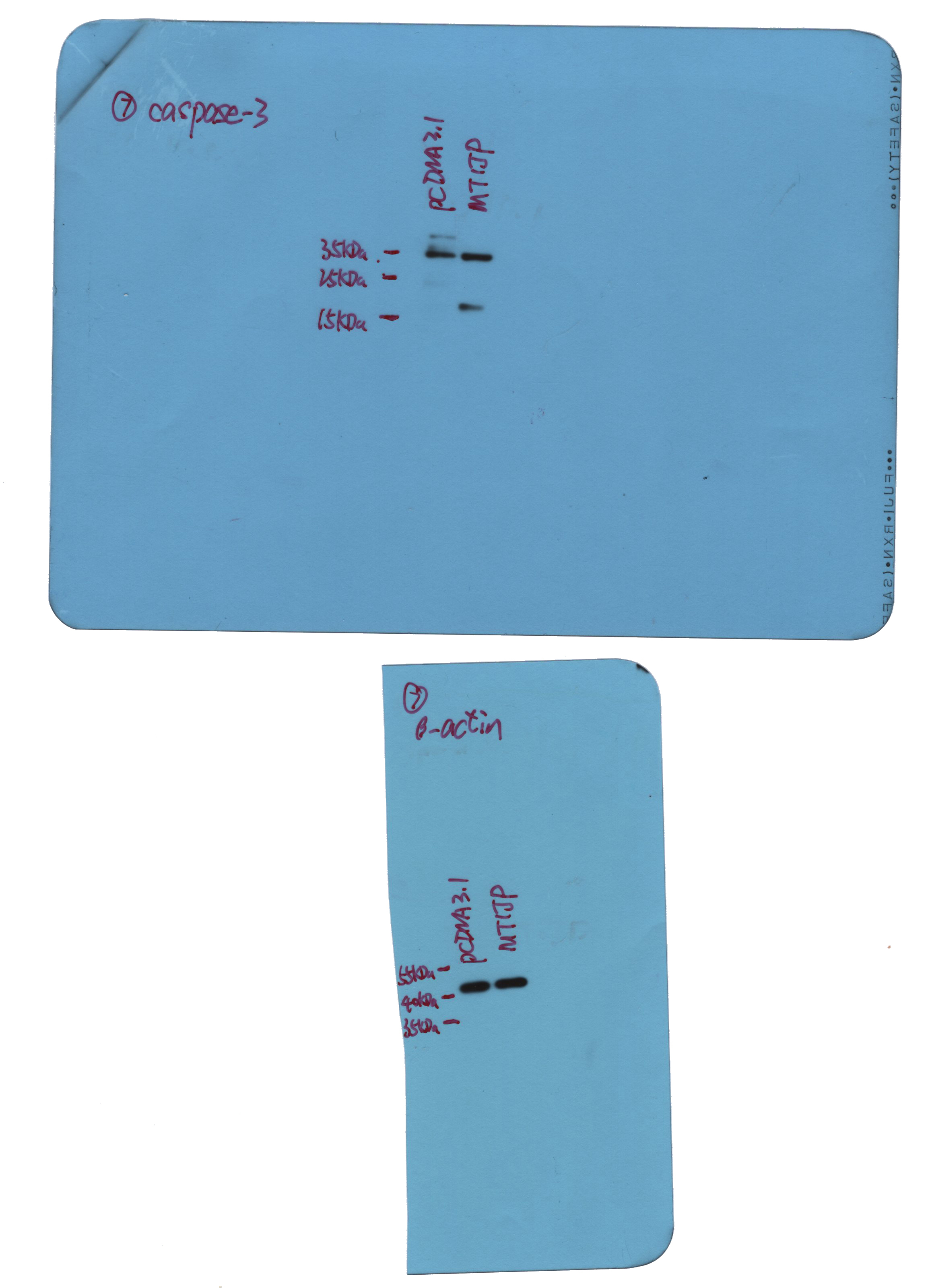

Supplement: Supplementary file 7 — Additional file 7 Fig. S7 The western blot bands of caspase-3 precursor, cleaved caspase-3 and the internal control after MT1JP overexpression in HCCC-9810 cells. [file 12885_2021_7838_MOESM7_ESM.jpg]

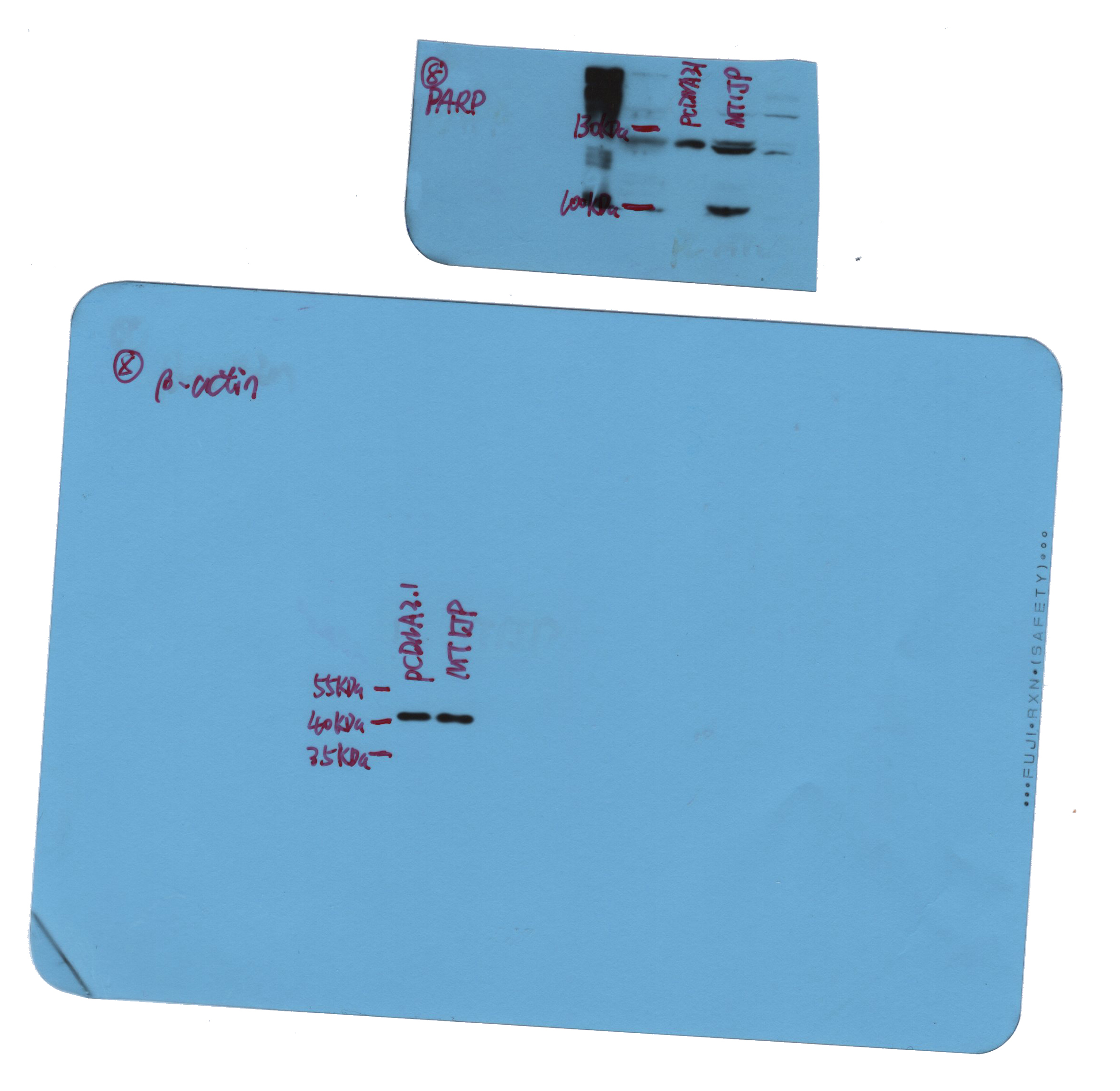

Supplement: Supplementary file 8 — Additional file 8 Fig. S8 The western blot bands of PARP precursor, cleaved PARP and the internal control after ectopic expression of MT1JP in HUCCT1 cells. [file 12885_2021_7838_MOESM8_ESM.jpg]

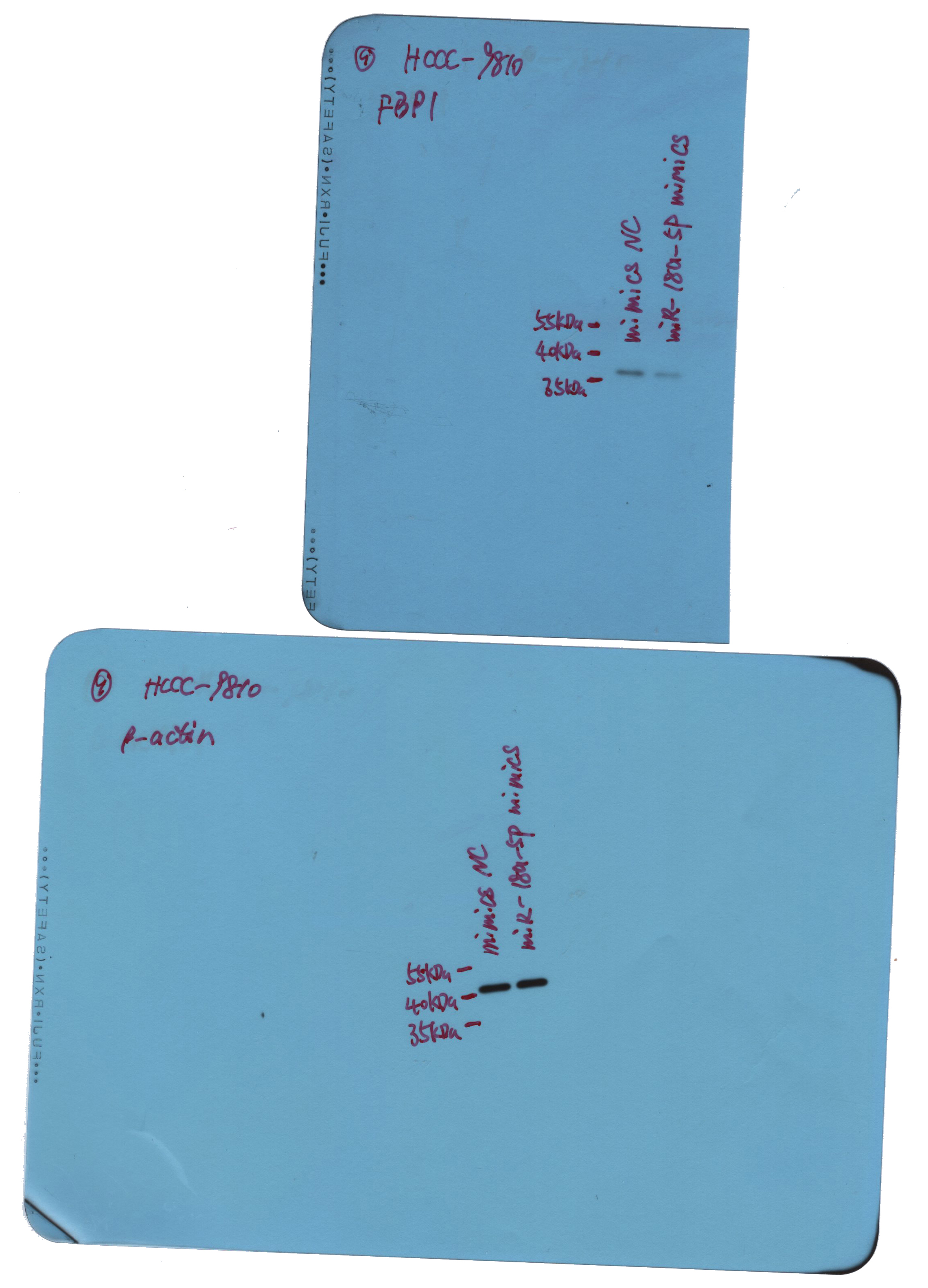

Supplement: Supplementary file 9 — Additional file 9 Fig. S9 The western blot bands of FBP1 and the internal control after transfection of miR-18a-5p mimics in HCCC-9810 cells. [file 12885_2021_7838_MOESM9_ESM.jpg]

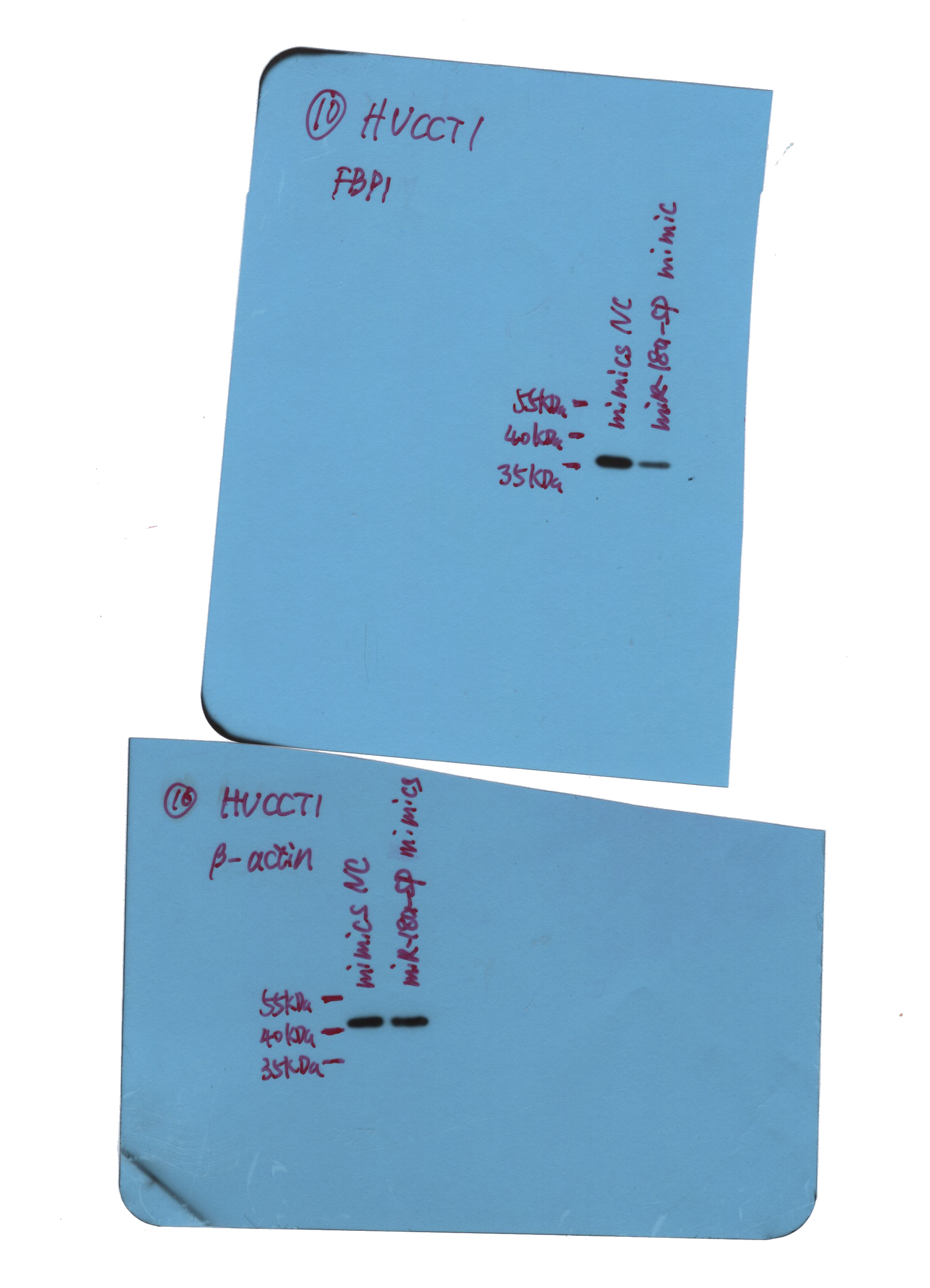

Supplement: Supplementary file 10 — Additional file 10 Fig. S10 The western blot bands of FBP1 and the internal control after overexpression of miR-18a-5p in HUCCT1 cells. [file 12885_2021_7838_MOESM10_ESM.jpg]

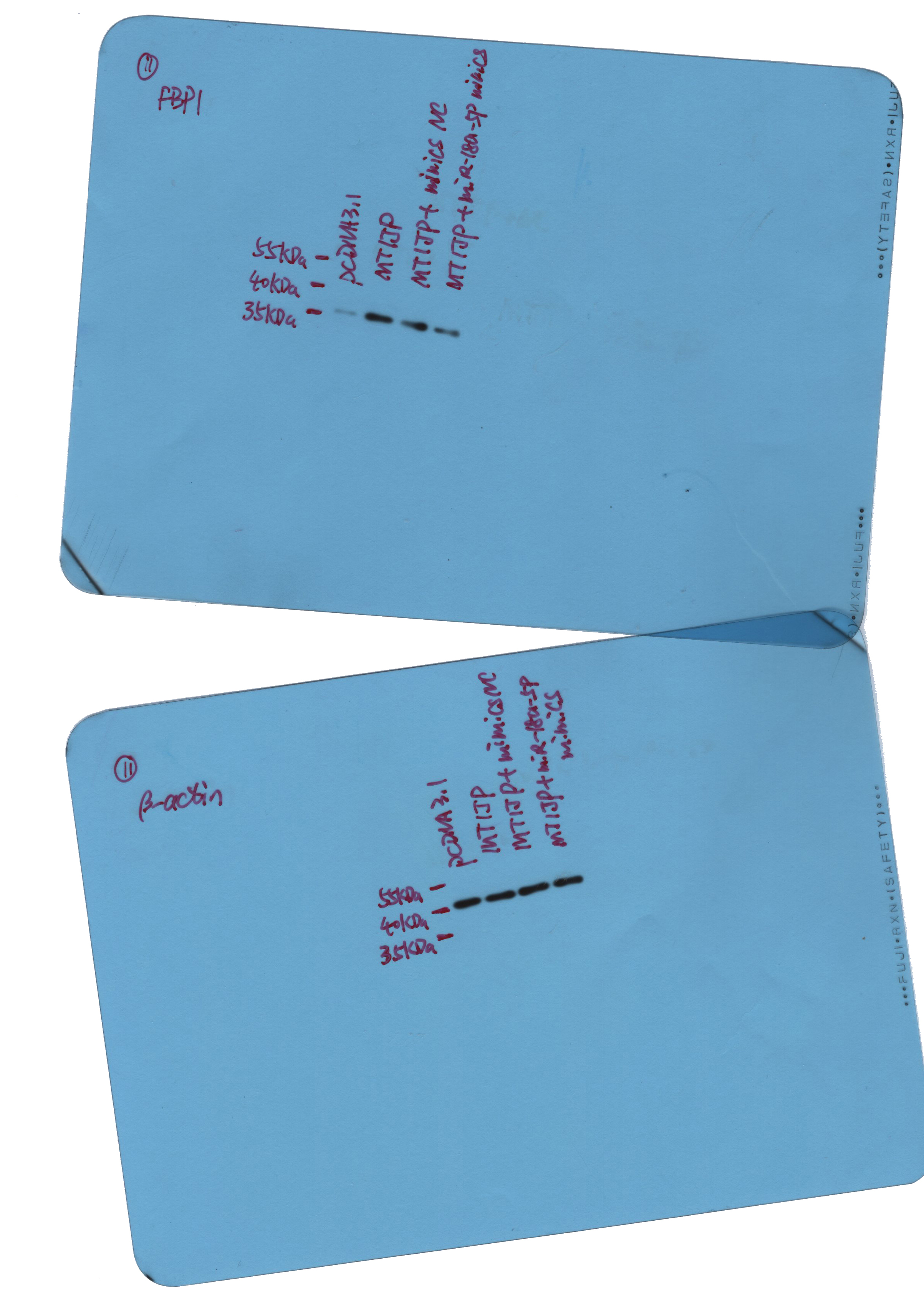

Supplement: Supplementary file 11 — Additional file 11 Fig. S11 The western blot bands of FBP1and the internal control after overexpression of MT1JP or/and miR-18a-5p. [file 12885_2021_7838_MOESM11_ESM.jpg]

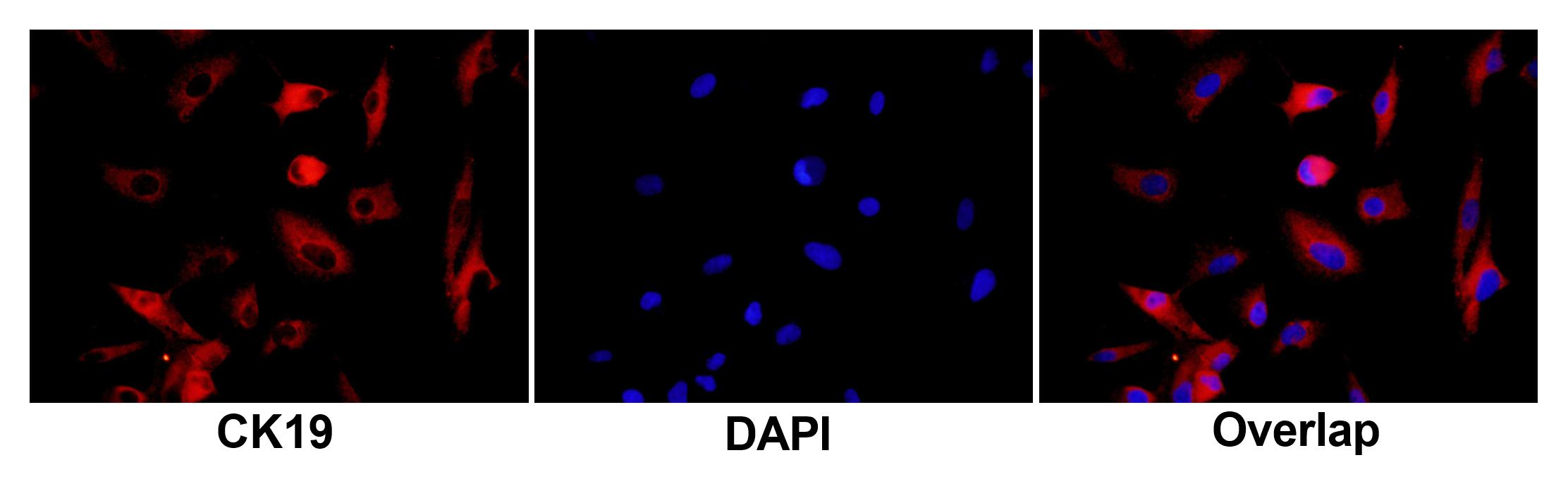

Supplement: Supplementary file 12 — Additional file 12 Fig. S12. Immunofluorescent staining of CK19 was used for identification of human primary intrahepatic cholangetic epithelial cells. [file 12885_2021_7838_MOESM12_ESM.jpg]
